# Agricultural activities and risk of central nervous system tumors among French farm managers: Results from the TRACTOR project

**DOI:** 10.1002/ijc.34197

**Published:** 2022-07-16

**Authors:** Pascal Petit, Gérald Gandon, Stéphan Chabardès, Vincent Bonneterre

**Affiliations:** ^1^ Univ. Grenoble Alpes, CNRS, UMR 5525, VetAgro Sup, Grenoble INP, CHU Grenoble Alpes, TIMC Grenoble France; ^2^ CHU Grenoble Alpes Occupational Diseases Center Grenoble France; ^3^ Univ. Grenoble Alpes, INSERM, U1216, CHU Grenoble Alpes, Grenoble Institut Neurosciences Grenoble France

**Keywords:** administrative health database, agriculture, cancer epidemiology, central nervous system tumor, health surveillance

## Abstract

The etiology of central nervous system (CNS) tumors is complex and involves many suspected risk factors. Scientific evidence remains insufficient, in particular in the agricultural field. The goal of our study was to investigate associations between agricultural activities and CNS tumors in the entire French farm manager workforce using data from the TRACTOR project. The TRACTOR project hold a large administrative health database covering the entire French agricultural workforce, over the period 2002‐2016, on the whole French metropolitan territory. Associations were estimated for 26 activities and CNS tumors using Cox proportional hazards model, with time to first CNS tumor insurance declaration as the underlying timescale, adjusting for sex, age and geographical area. There were 1017 cases among 1 036 069 farm managers, including 317 meningiomas and 479 gliomas. Associations varied with tumor types, sex and types of crop and animal farming. Analyses showed several increased risks of CNS tumors, in particular for animal farming. The main increases in risk were observed for meningioma in mixed dairy and cow farming (hazard ratio [HR] = 1.75, 95% confidence interval [CI]: 1.09‐2.81) and glioma in pig farming (HR = 2.28, 95% CI: 1.37‐3.80). Our study brings new insights on the association of a wide range of agricultural activities and CNS tumor and subtype‐specific risks in farm managers. Although these findings need to be corroborated in further studies and should be interpreted cautiously, they could have implications for enhancing CNS tumor surveillance in agriculture.

## What's new?

Cancers of the central nervous system (CNS) are linked to certain agricultural activities, including animal farming and pesticide use. Whether these agricultural activities have a role in CNS cancer etiology, however, remains uncertain. Here, among farm workers in France, 26 agricultural activities were investigated for potential associations with risk of CNS tumors, including type‐specific CNS cancers. CNS cancer risk varied in association with sex and types of farming. In particular, farming of specific animals increased risk of several CNS cancers, including meningioma and glioma. While further research is needed, the findings may have implications for CNS tumor surveillance in agriculture.

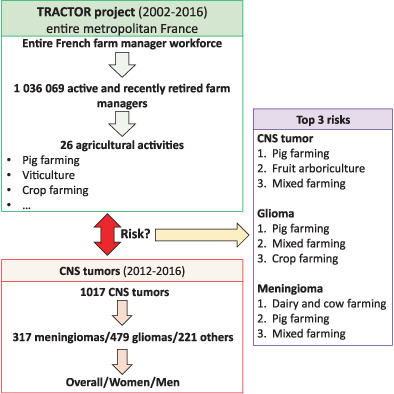

**Abbreviations**
95% CI: 95% confidence interval
CNIL: independent administrative authority protecting privacy and personal data
CNS: central nervous system
HR: hazard ratio
ICD‐10: 10th revision of the International Statistical Classification of Diseases and Related Health Problems
MSA: National Health Insurance Fund for Agricultural Workers and Farmers
OR: odds ratio
TRACTOR: tracking and monitoring occupational risks in agriculture
VIF: variance inflation factor

## 1 INTRODUCTION

The incidence of central nervous system (CNS) cancers has increased worldwide from 17% in the last two decades.^1^ The etiology of the CNS tumors remains mostly unknown and few risk factors have been identified. A synthesis of 40 years of epidemiologic studies of farming and brain cancer concluded that farming (as a large entity), livestock farming and “documented exposure to pesticides” were associated with increased risks of CNS tumors.^2^ However, results about the involvement of pesticides in the occurrence of CNS tumors are still scarce, inconsistent and insufficient.^3^, ^4^, ^5^

There are limited cohort studies investigated work‐related cancer in agriculture.^6^ In France, the AGRICAN cohort includes about 180 000 people living in 11 French metropolitan counties beneficiating from cancer registries.^7^, ^8^, ^9^ AGRICAN includes only 7% of all active French agricultural workers covered by the National Health Insurance Fund for Agricultural Workers and Farmers (MSA) data (retired persons excluded). Other French studies are also limited both geographically and in scope, and pertained only to a small proportion of the agricultural workforce.^10^, ^11^, ^12^ It is therefore paramount to consolidate existing and recent evidences by studying the entire French agricultural workforce on the whole metropolitan territory.

Data on the entire French agricultural workforce are available to the TRACTOR project.^13^ The goal of our study was to investigate the associations between agricultural activities and CNS tumors in the entire French farm manager workforce over the period 2002‐2016 on the whole French metropolitan territory.

## 2 MATERIALS AND METHODS

### 2.1 Population

We selected in our analysis all farm managers, including farm or company managers, owners and self‐employed persons, from 2002 to 2016 within the TRACTOR project. The study population have been described previously.^13^ Briefly, yearly routinely collected insurance data on contributor' demographic characteristics and health are available for the TRACTOR project. Demographic characteristics (eg, occupation, age, sex, farm surface) are collected by MSA from forms that are filled by farm managers during their yearly insurance affiliation. Each occupational activity is then coded by MSA according to an internal thesaurus referring to 26 different activities: truck farming, floriculture/flower‐growing; fruit arboriculture; garden center; crop farming (including field crops, cereal grain crops, wheat and industrial grower); viticulture; sylviculture/forestry; unspecified specialized crop farming (eg, horticulture); dairy farming (individuals performing only dairy farming); cow farming (individuals performing only cow farming); both/mixed dairy and cow farming (individuals performing both dairy and cow farming); ovine and caprine farming; pig farming; stud farming; unspecified large animal farming (eg, large dogs, zoo); poultry and rabbit farming; unspecified small animal farming (eg, frogs, snails, bees); training, dressage, riding clubs; unspecified and mixed farming (eg, polyculture, mixed farming); shellfish farming; salt marsh; wood production; fixed sawmill; agricultural work companies; gardening, landscaping and reforestation companies; company representative/authorized representative; rural craftsperson. These activities refer to the main activity in terms of effective working time and only allowed for an indirect exposure estimation/ascertainment. As for health data, they pertained to chronic diseases/long‐term illnesses for which farm managers are entitled to fee exemption and the full coverage of health care expenditures between 2012‐2016.

### 2.2 CNS cancer identification and statistical analysis

CNS tumor cases were identified using ICD‐10 codes (10th revision of the International Statistical Classification of Diseases and Related Health Problems). Information on CNS tumor cases came from administrative insurance health data (MSA), where each disease is coded by MSA insurance physicians with a 3‐digit long ICD‐10 code based on patient medical reports.^13^ Table 1 presents all ICD‐10 codes and grouping of ICD‐10 codes considered in this work.

**TABLE 1:** ICD‐10 codes and grouping of ICD‐10 codes for identifying CNS tumors

Designation in this paper	ICD‐10 codes	Definition
CNS tumors	C70, C71, C72, D32, D33, D42, D43	All benign and malignant CNS tumors
Overall meningiomas	C70, D32, D42	Both malignant and benign meningiomas
Malignant meningiomas	C70	Malignant neoplasm of meninges
Malignant gliomas	C71	Malignant neoplasm of brain
Other CNS tumors	C72	Malignant neoplasm of spinal cord, cranial nerves and other parts of CNS
Benign meningiomas	D32	Benign neoplasm of meninges
Benign gliomas and other CNS tumors	D33	Benign neoplasm of brain and other parts of CNS
Uncertain meningiomas	D42	Neoplasm of uncertain or unknown behavior of meninges
Uncertain gliomas and other CNS tumors	D43	Neoplasm of uncertain or unknown behavior of brain and central nervous system

Abbreviations: CNS, central nervous system; ICD‐10, 10th revision of the International Statistical Classification of Diseases and Related Health Problems.

To assess CNS tumor risk related to agricultural activities, hazard ratios (HRs) and 95% confidence intervals (CIs) were estimated using Cox proportional hazards model, with time to first CNS tumor insurance declaration as the underlying timescale. The reference group included farm managers who did not carry out the activity of interest. For instance, for pig farmers, the reference group included every farm managers that did not farm pigs between 2002 and 2016 and could therefore include individuals who may be exposed to pesticides or other risk factors. CNS tumors risks (overall and by types) were estimated according to each of the 26 activities when the number of exposed cases exceeded or equaled 3. Only the main agricultural activity in terms of effective working time was known.^13^

All analyses were adjusted for age (<40, 40‐49, 50‐59, ≥60) and sex. We also conducted analyses stratified by sex to identify potential gender specific CNS tumor risks that may come from differences in occupational exposure and tasks between women and men. Several potential confounders (covariates) were considered (Table 2). The selection of covariates for the Cox proportional hazards model was based on the variance inflation factor (VIF).^14^ Collinear covariates, with a VIF > 2.5, were not included in the models. All analyses were also adjusted for the 13 metropolitan French administrative geographical regions where the farm is located to account for a potential confounding effect related to possible unmeasured and unequally distributed geographically risk factors. Some administrative geographic regions could be correlated with agricultural activities, which may mask associations with exposures. To address this matter, we applied a restrictive variable selection based on the VIF (≤2.5). Therefore, only administrative geographic regions poorly or not collinear with agricultural activities have been included in each model. Hence, depending on the model considered, different administrative geographic regions could be taken into account and, for some models, it could sometimes happened that no administrative geographic regions were considered if they were all found to be collinear (VIF > 2.5) with the activity of interest. No methods to handle missing data was needed because data originated from compulsory agricultural insurance fund, which was complete for all variables of interest available to the TRACTOR project. All statistical analyses were performed using R software 4.1.2 (R Core Team, Vienna, Austria) for Windows 10©.

**TABLE 2:** List of potential covariates considered in the analyses

Covariate	Modality
First year of the farm's establishment	4 categories: <1985, 1985‐1994, 1995‐2004, >2004
Farm surface (expressed in 100 square meters)	5 categories: 0, ]0‐500[, [500‐2500[, [2500‐5000[, ≥5000
Median yearly earnings (in euros)^a^	5 categories: <0, [0‐1500[, [1500‐5000[, [5000‐10 000[, ≥10 000
Number of associates	3 categories: 0, 1, >1
Unemployment status	2 categories: never unemployed or had been unemployed at least once over the period 2002‐2016
Number of farms	2 categories: 1 or >1
Family status	2 categories: single or as a couple
Partner work status	2 categories: perform or do not perform task to help farm manager
Having a secondary activity	2 categories: yes or no
Number of comorbidities	3 categories: 0, 1, >1
Geographical regions	13 categories: 13 metropolitan French administrative geographical areas

^a^ Income part that is taken into account for insurance contribution.

## 3 RESULTS

### 3.1 Population characteristics

Baseline characteristics of the study population are presented in Table 3. Around one third of all farm managers were crop farmers (29.5%), while 15.3% and 11.4% performed dairy farming and viticulture activities, respectively (Figure S1). Among the 1 036 069 farm managers available to TRACTOR over the period 2002‐2016, a total of 1017 (0.1%) had a CNS tumor declaration. The proportion of women was higher for farm managers with a CNS tumor than without (39% vs 31%). Overall, farm managers with a CNS tumor were older than farm managers without a CNS tumor (median age of 59 years old vs 56 years old), established their farm in earlier time periods, had a bigger farm surface (median of 3092 hundred square meters vs 1699) and a higher number of comorbidities (20% vs 15%).

**TABLE 3:** Baseline characteristics of the study population, TRACTOR project, France, 2002‐2016

	Farm manager without CNS tumors (n = 1 035 052)	Farm manager with CNS tumors (n = 1017)
Main characteristics	n (%)	n (%)
Sex		
Female	319 993 (31)	399 (39)
Male	715 059 (69)	618 (61)
Age group (years)		
<40	151 015 (15)	56 (6)
40‐49	185 940 (18)	108 (11)
50‐59	315 721 (31)	366 (36)
≥60	382 376 (37)	487 (48)
Family status		
Single	437 574 (42)	278 (28)
As a couple	597 478 (58)	739 (73)
First year of the farm's establishment		
<1985	105 957 (10)	130 (13)
1985‐1994	439 892 (42)	519 (51)
1995‐2004	255 128 (25)	238 (23)
>2004	238 912 (23)	138 (13)
Farm surface (expressed in 100 square meters)		
0	110 425 (11)	58 (6)
]0‐500[	223 910 (22)	144 (14)
[500‐2500[	255 700 (25)	245 (24)
[2500‐5000[	190 024 (18)	240 (24)
5000 and more	254 993 (25)	330 (32)
Farm location (region)		
Auvergne‐Rhône‐Alpes	112 505 (10.9)	101 (9.9)
Bourgogne‐Franche‐Comté	63 238 (6.1)	59 (5.8)
Bretagne	77 054 (7.5)	88 (8.7)
Centre—Val de Loire	47 412 (4.6)	58 (5.7)
Corse	5053 (0.5)	3 (0.3)
Grand Est	79 488 (7.7)	66 (6.5)
Hauts‐de‐France	46 130 (4.5)	41 (4.0)
Île‐de‐France	13 546 (1.3)	15 (1.5)
Normandie	76 759 (7.4)	89 (8.8)
Nouvelle‐Aquitaine	171 625 (16.6)	192 (18.9)
Occitanie	158 153 (15.3)	136 (13.4)
Provence‐Alpes‐Côte d'Azur	105 102 (10.2)	91 (9.0)
Pays de la Loire	78 987 (7.6)	78 (7.7)
Number of farms		
1 farm	1 001 302 (97)	984 (97)
>1 farm	33 750 (3)	33 (3)
Partner work status		
Do not perform task to help farm manager	945 273 (91)	876 (86)
Perform task to help farm manager	89 779 (9)	141 (14)
Number of associates		
0	783 088 (76)	736 (72)
≥1	251 964 (24)	281 (28)
Secondary activity		
No secondary activity	723 088 (70)	910 (90)
At least one secondary activity	311 964 (30)	107 (10)
Median yearly earnings (euros)^a^		
<0	35 589 (4)	29 (3)
[0‐1500[	272 498 (26)	146 (14)
[1500‐5000[	198 445 (19)	200 (20)
[5000‐10 000[	234 737 (23)	271 (27)
>10 000	293 783 (28)	371 (37)
Unemployment status		
Never unemployed	1 022 369 (99)	1012 (99)
Had been unemployed at least once	12 683 (1)	5 (1)
Number of comorbidities		
0	882 606 (85)	810 (80)
1	106 191 (10)	162 (16)
>1	46 255 (5)	45 (4)

^a^ Income part that is taken into account for insurance contribution.

Over 58% of the CNS cases were malignant neoplasms (Table 4). Most CNS tumors were gliomas (47%). Gliomas affected more men (57%) than women (32%). A total of 32 (3.1%) individuals (13 men and 19 women) were declared with several types of ICD‐10 codes for CNS cancers. The percentages of CNS tumors varied depending on the agricultural practice/activity and sex (Figure S2).

**TABLE 4:** Number of CNS tumors, by sex and type, TRACTOR project, France, 2002‐2016

Cancer type	ICD‐10 code	Both sexes, n (%)	Female, n (%)	Male, n (%)
All CNS tumors	C70, C71, C72, D32, D33, D42, D43	1017 (100)	399 (100)	618 (100)
Meningiomas (all)	C70, D32, D42	317 (31)	191 (48)	126 (20)
Malignant meningiomas	C70	71 (7.0)	41 (10.3)	30 (4.9)
Benign meningiomas	D32	154 (15)	100 (25)	54 (8.7)
Uncertain meningiomas	D42	108 (10.6)	62 (16)	46 (7.4)
Malignant gliomas	C71	479 (47)	126 (32)	353 (57)
Benign gliomas and other CNS tumors	D33	76 (7.5)	24 (6.0)	52 (8.4)
Uncertain gliomas and other CNS tumors	D43	110 (10.8)	38 (9.5)	72 (12)
Other CNS tumors	C72	42 (4.1)	18 (4.5)	24 (3.9)

*Note*: Refer to Table 1 for ICD‐10 code definitions.

Abbreviation: CNS, central nervous system.

### 3.2 Risk associated with agricultural activities

Associations varied with CNS tumor types, sex and types of crop and animal farming (Table 5 for results adjusted for sex and Figures S3‐S5 and Tables S1 and S2 in the Supplemental Materials for results stratified by sex). Analyses showed several increased risks of CNS tumors, in particular for animal farming. Results for all CNS tumors (regardless of subtypes) are presented in Tables 5 and S1 for comparison purposes with the literature, but will not be commented further as it is more relevant to focus on the most accurate disease description the data allowed.

**TABLE 5:** Agricultural practices and risks of CNS tumors, TRACTOR, France, 2002‐2016

Agricultural practice/activity	Study population (%)	All CNS tumors	All meningiomas	C70	D32	D42	C71	D33	D43	C72
		HR^a^ [95% CI]; *m* ^b^ (%)	HR^a^ [95% CI]; *m* ^c^ (%)	HR^a^ [95% CI]; *m* ^c^ (%)	HR^a^ [95% CI]; *m* ^c^ (%)	HR^a^ [95% CI]; *m* ^c^ (%)	HR^a^ [95% CI]; *m* ^c^ (%)	HR^a^ [95% CI]; *m* ^c^ (%)	HR^a^ [95% CI]; *m* ^c^ (%)	HR^a^ [95% CI]; *m* ^c^ (%)
Both/mixed dairy and cow farming (individuals performing both dairy and cow farming)	30 729 (3.0)	0.98 [0.70‐1.37]; 36 (3.5)	**1.75 [1.09‐2.81]**; 19 (52.7)	1.62 [0.64‐4.09]; 5 (13.9)	0.91 [0.37‐2.24]; 5 (13.9)	**2.67 [1.32‐5.39]**; 9 (25.0)	0.62 [0.34‐1.13]; 11 (30.6)	NC; 1 (2.8)	1.24 [0.50‐3.10]; 5 (13.9)	NC; 0 (0)
Cow farming (individuals performing only cow farming)	110 214 (10.6)	1.03 [0.85‐1.25]; 121 (11.9)	1.30 [0.94‐1.79]; 46 (38.0)	1.05 [0.51‐2.14]; 9 (7.4)	**1.69 [1.11‐2.56]**; 29 (24.0)	0.72 [0.36‐1.45]; 9 (7.4)	0.78 [0.57‐1.07]; 45 (37.2)	0.63 [0.29‐1.39]; 7 (5.8)	**1.84 [1.11‐3.02]**; 21 (17.4)	NC; 2 (1.7)
Dairy farming (individuals performing only dairy farming)	158 706 (15.3)	1.04 [0.87‐1.23]; 186 (18.3)	0.78 [0.56‐1.10]; 45 (24.2)	1.25 [0.69‐2.24]; 18 (9.7)	0.74 [0.45‐1.21]; 20 (10.8)	0.62 [0.33‐1.18]; 12 (6.5)	1.18 [0.92‐1.51]; 99 (53.2)	1.53 [0.84‐2.81]; 16 (8.6)	0.77 [0.43‐1.37]; 16 (8.6)	**2.10 [1.01‐4.34]**; 13 (7.0)
Ovine and caprine farming	47 086 (4.5)	1.17 [0.86‐1.58]; 45 (4.4)	1.47 [0.88‐2.45]; 16 (35.6)	NC; 2 (4.4)	1.10 [0.48‐2.52]; 6 (13.3)	**2.27 [1.08‐4.76]**; 8 (17.8)	1.26 [0.82‐1.93]; 23 (51.1)	1.23 [0.44‐3.40]; 4 (8.9)	0.77 [0.28‐2.14]; 4 (8.9)	NC; 0 (0)
Pig farming	13 389 (1.3)	**1.67 [1.10‐2.54];** 24 (2.4)	1.58 [0.68‐3.64]; 6 (25.0)	NC; 1 (4.2)	NC; 1 (4.2)	**3.18 [1.11‐9.16]**; 4 (16.7)	**2.28 [1.37‐3.80]**; 17 (70.8)	NC; 0 (0)	NC; 0 (0)	NC; 1 (4.2)
Poultry and rabbit farming	24 576 (2.4)	1.13 [0.76‐1.69]; 25 (2.5)	0.93 [0.41‐2.09]; 6 (24.0)	NC; 1 (4.0)	NC; 2 (8.0)	1.36 [0.42‐4.34]; 3 (12.0)	1.14 [0.65‐2.00]; 13 (52.0)	1.72 [0.53‐5.55]; 3 (12.0)	NC; 2 (8.0)	NC; 2 (8.0)
Stud farming	15 641 (1.5)	0.85 [0.38‐1.91]; 6 (0.6)	1.53 [0.49‐4.81]; 3 (50.0)	NC; 0 (0)	3.16 [0.99‐10.0]; 3 (50.0)	NC; 0 (0)	0.84 [0.27‐2.62]; 3 (50.0)	NC; 0 (0)	NC; 0 (0)	NC; 0 (0)
Training, dressage, riding clubs	13 273 (1.3)	0.91 [0.45‐1.83]; 8 (0.8)	NC; 2 (25.0)	NC; 1 (12.5)	NC; 0 (0)	NC; 1 (12.5)	0.83 [0.31‐2.24]; 4 (50.0)	NC; 0 (0)	NC; 2 (25.0)	NC; 0 (0)
Unspecified large animal farming (eg, large dogs, zoo)	2663 (0.3)	**3.67 [1.37‐9.82];** 4 (0.4)	NC; 2 (50.0)	NC; 1 (25.0)	NC; 1 (25.0)	NC; 0 (0)	NC; 1 (25.0)	NC; 0 (0)	NC; 1 (25.0)	NC; 0 (0)
Unspecified small animal farming (eg, frogs, snails, bees)	18 058 (1.7)	1.56 [0.84‐2.91]; 10 (1.0)	1.54 [0.49‐4.80]; 3 (30.0)	NC; 0 (0)	NC; 2 (20.0)	NC; 1 (10.0)	1.31 [0.49‐3.52]; 4 (40.0)	NC; 1 (10.0)	NC; 2 (20.0)	NC; 0 (0)
Fruit arboriculture	24 086 (2.3)	**1.50 [1.02‐2.21];** 27 (2.7)	0.5 [0.16‐1.56]; 3 (11.1)	NC; 0 (0)	NC; 2 (7.4)	NC; 1 (3.7)	**1.72 [1.00‐2.94]**; 14 (51.9)	2.11 [0.66‐6.79]; 3 (11.1)	**3.05 [1.31‐7.08]**; 6 (22.2)	NC; 2 (7.4)
Garden center	5111 (0.5)	0.87 [0.28‐2.70]; 3 (0.3)	NC; 2 (66.7)	NC; 0 (0)	NC; 2 (66.7)	NC; 0 (0)	NC; 0 (0)	NC; 1 (33.3)	NC; 0 (0)	NC; 0 (0)
Truck farming, floriculture/flower‐growing	41 525 (4.0)	**1.36 [1.00‐1.84];** 44 (4.3)	1.46 [0.85‐2.51]; 14 (31.8)	1.79 [0.56‐5.73]; 3 (6.8)	1.69 [0.82‐3.48]; 8 (18.2)	0.87 [0.27‐2.76]; 3 (6.8)	1.08 [0.66‐1.76]; 17 (38.6)	1.11 [0.35‐3.56]; 3 (6.8)	1.52 [0.66‐3.51]; 6 (13.6)	**3.63 [1.27‐10.4]**; 4 (9.1)
Unspecified and mixed farming (eg, polyculture, mixed farming)	120 746 (11.7)	**1.34 [1.12‐1.61];** 140 (13.8)	1.10 [0.77‐1.56]; 36 (25.7)	0.88 [0.40‐1.94]; 7 (5.0)	0.72 [0.40‐1.31]; 12 (8.6)	**1.83 [1.11‐3.03]**; 20 (14.3)	**1.52 [1.17‐1.96]**; 72 (51.4)	1.47 [0.80‐2.70]; 13 (9.3)	1.48 [0.87‐2.52]; 17 (12.1)	1.04 [0.43‐2.53]; 6 (4.3)
Unspecified specialized crop farming (eg, horticulture)	6168 (0.6)	1.21 [0.45‐3.23]; 4 (0.4)	NC; 2 (50.0)	NC; 0 (0)	NC; 2 (50.0)	NC; 0 (0)	NC; 1 (25.0)	NC; 1 (25.0)	NC; 0 (0)	NC; 0 (0)
Viticulture	118 577 (11.4)	1.21 [0.98‐1.48]; 113 (11.1)	1.31 [0.92‐1.85]; 40 (35.4)	0.97 [0.41‐2.33]; 6 (5.3)	1.59 [0.99‐2.55]; 22 (19.5)	1.15 [0.62‐2.11]; 13 (11.5)	1.29 [0.95‐1.74]; 53 (46.9)	1.49 [0.78‐2.84]; 12 (10.6)	0.70 [0.33‐1.47]; 8 (7.1)	NC; 1 (0.9)
Crop farming (including field crops, cereal grain crops, wheat and industrial grower)	305 838 (29.5)	**1.20 [1.03‐1.41];** 258 (25.4)	1.13 [0.86‐1.48]; 87 (33.7)	1.43 [0.80‐2.55]; 19 (7.4)	1.18 [0.81‐1.73]; 46 (17.8)	1.05 [0.66‐1.67]; 29 (11.2)	**1.28 [1.01‐1.61]**; 116 (45.0)	**1.64 [1.00‐2.69]**; 29 (11.2)	1.04 [0.62‐1.76]; 21 (8.1)	1.66 [0.77‐3.59]; 11 (4.3)
Agricultural work companies	14 282 (1.4)	1.04 [0.58‐1.90]; 11 (1.1)	1.35 [0.50‐3.62]; 4 (36.4)	NC; 0 (0)	NC; 1 (9.1)	3.05 [0.96‐9.68]; 3 (27.3)	0.77 [0.29‐2.07]; 4 (36.4)	NC; 2 (18.2)	NC; 1 (9.1)	NC; 0 (0)
Company representative/authorized representative	1846 (0.2)	**4.15 [1.33‐12.9];** 3 (0.3)	NC; 1 (33.3)	NC; 0 (0)	NC; 1 (33.3)	NC; 0 (0)	NC; 1 (33.3)	NC; 0 (0)	NC; 1 (33.3)	NC; 0 (0)
Gardening, landscaping and reforestation companies	44 948 (4.3)	0.87 [0.59‐1.28]; 28 (2.8)	0.59 [0.24‐1.45]; 5 (17.9)	2.23 [0.68‐7.26]; 3 (10.7)	NC; 2 (7.1)	NC; 0 (0)	0.80 [0.45‐1.41]; 13 (46.4)	1.41 [0.49‐4.04]; 4 (14.3)	0.93 [0.36‐2.40]; 5 (17.9)	NC; 1 (3.6)
Wood production	10 470 (1.0)	0.83 [0.44‐1.55]; 10 (1.0)	NC; 2 (20.0)	NC; 0 (0)	NC; 1 (10.0)	NC; 1 (10.0)	1.08 [0.48‐2.43]; 6 (60.0)	NC; 0 (0)	NC; 1 (10.0)	NC; 1 (10.0)
Shellfish farming	3350 (0.3)	NC; 2 (0.2)	NC; 0 (0)	NC; 0 (0)	NC; 0 (0)	NC; 0 (0)	NC; 2 (100)	NC; 0 (0)	NC; 0 (0)	NC; 0 (0)
Salt marsh	873 (0.08)	NC; 0 (0)	NC; 0 (0)	NC; 0 (0)	NC; 0 (0)	NC; 0 (0)	NC; 0 (0)	NC; 0 (0)	NC; 0 (0)	NC; 0 (0)
Fixed sawmill	735 (0.07)	NC; 0 (0)	NC; 0 (0)	NC; 0 (0)	NC; 0 (0)	NC; 0 (0)	NC; 0 (0)	NC; 0 (0)	NC; 0 (0)	NC; 0 (0)
Rural craftsperson	7038 (0.7)	NC; 0 (0)	NC; 0 (0)	NC; 0 (0)	NC; 0 (0)	NC; 0 (0)	NC; 0 (0)	NC; 0 (0)	NC; 0 (0)	NC; 0 (0)
Sylviculture/forestry	1986 (0.2)	NC; 2 (0.2)	NC; 0 (0)	NC; 0 (0)	NC; 0 (0)	NC; 0 (0)	NC; 2 (100)	NC; 0 (0)	NC; 0 (0)	NC; 0 (0)

*Note*: Bolded values indicate increased (when the lower bound of the 95% CI is >1) and decreased (when the upper bound of the 95% CI is <1) risks of CNS tumors.

Abbreviations: 95% CI, 95% confidence interval; C70, malignant neoplasm of meninges; C71, malignant neoplasm of brain; C72, malignant neoplasm of spinal cord, cranial nerves and other parts of CNS; CNS, central nervous system; D32, benign neoplasm of meninges; D33, benign neoplasm of brain and other parts of CNS; D42, neoplasm of uncertain or unknown behavior of meninges; D43, neoplasm of uncertain or unknown behavior of brain and central nervous system; HR, hazard ratio; *m*, number of exposed cases; NC, not calculated.

^a^ Hazard ratios were estimated by Cox models with time to first CNS tumor insurance declaration as the underlying timescale, when the number of exposed cases was sufficient (*m* ≥ 3), adjusted for sex (for “both sexes” only), age, first year of the farm's establishment, farm surface, earnings, number of associates, unemployment status, total number of farms, family status, partner work status, farm location, number of comorbidities and having a secondary activity.

^b^ The percentages in brackets refer to the ratio of exposed cases in the study population and the total number of cases in the overall population.

^c^ The percentages in brackets refer to the ratio of exposed cases in the study population and the total number of cases in the study population.

Regarding meningiomas, for “both sexes” (results adjusted on sex), ovine and caprine farming was associated with increased risks of uncertain meningiomas (HR = 2.27 [1.08‐4.76]) and stud farming with positive trends for benign meningiomas (HR = 3.16 [0.99‐10]) (Tables 5 and S1). Mixed dairy and cow farming was associated with increased risks of overall meningiomas (HR = 2.69 [1.46‐4.97]) and uncertain meningiomas (HR = 4.83 [1.95‐12]) in men. Cow farming (HR = 1.68 [1.01‐2.79]) and dairy farming (HR = 0.52 [0.26‐1.04]) were, respectively, associated with increased risks and negative trends of benign meningiomas in women. Pig farming (HR = 8.11 [2.29‐28.7]) and agricultural work companies (HR = 5.46 [1.66‐18]) were associated with increased risks of uncertain meningiomas in men. Women involved in unspecified and mixed farming had increased risks of uncertain meningiomas (HR = 2.34 [1.26‐4.35]). Viticulture was associated with positive trends regarding the risks of overall meningiomas (HR = 1.49 [0.96‐2.28]) and benign meningiomas (HR = 1.59 [0.99‐2.55]) in women.

Regarding malignant gliomas, increased risks were found in pig farming (HR = 2.52 [1.43‐4.45]) and unspecified and mixed farming (HR = 1.47 [1.09‐1.98]) for men (Tables 5 and S2). Dairy farming was associated with increased risks in men (HR = 1.43 [1.08‐1.89]) but with decreased risks in women (HR = 0.57 [0.33‐0.98]) as well as mixed dairy and cow farming in men (HR = 0.43 [0.19‐0.97]). Poultry and rabbit farming (HR = 2.31 [1.10‐4.85]) and fruit arboriculture (HR = 2.58 [1.12‐5.96]) were associated with increased risks for women. Crop farming (HR = 1.22 [0.93‐1.59]) and ovine and caprine farming (HR = 1.55 [0.96‐2.49]) were associated with positive trends in men.

## 4 DISCUSSION

In our study, increases in the risk of CNS tumors were observed in relation to various occupational agricultural activities performed by French farm managers. Associations varied with tumor types and kinds of crop and animal farming, suggesting that part of the risk could be attributable to agricultural activities. In addition, for a given agricultural activity, associations could vary by sex, suggesting that differences in occupational exposure and tasks, sometimes gender specific, could contribute to the differences in risks between women and men.

### 4.1 Risks associated with agricultural activities

Several studies have examined agricultural exposure and the risk of CNS tumors. Results from the literature are inconsistent, in particular for overall agriculture, with for instance several studies that found an increased risk of CNS tumors related to agriculture,^2^, ^10^ while others found a decreased risk.^2^ Most studies focused on overall CNS tumors, without giving information on specific types such as gliomas or meningiomas. The AGRICAN cohort was the most detailed study found in the literature regarding agricultural exposure and the risk of CNS tumors.^7^, ^8^, ^9^

**FIGURE 1:**
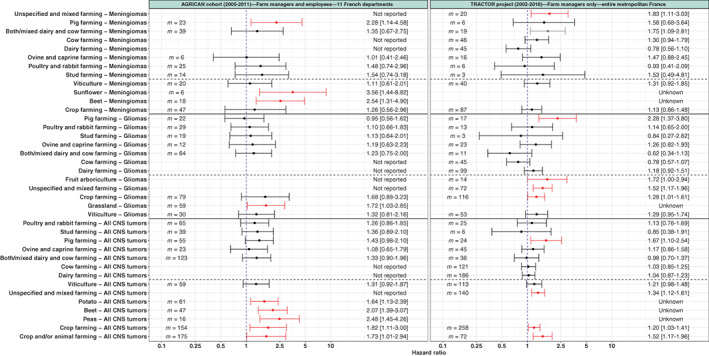
Agricultural practices and risks of CNS tumors—comparison between the AGRICAN cohort study and the TRACTOR project. *m*, number of cancer cases. Results from AGRICAN are extracted from Piel et al.^7^ Regarding the AGRICAN study, hazard ratios were estimated by Cox models with age as underlying timescale, adjusted for sex, educational level (not for meningiomas), smoking (both status and number of pack years centered, not for gliomas), alcohol consumption (not for meningiomas) and coexposures between farming types. Regarding the TRACTOR project, hazard ratios were estimated by Cox models with time to first CNS tumor insurance declaration as the underlying timescale, when the number of exposed cases was sufficient (*m* ≥ 3), adjusted for sex, age, first year of the farm's establishment, farm surface, earnings, number of associates, unemployment status, total number of farms, family status, partner work status, farm location, number of comorbidities and having a secondary activity (Table 2) [Color figure can be viewed at wileyonlinelibrary.com]

A detailed comparison between results from the French cohort AGRICAN and our study is presented in Figure 1. There were some differences between AGRICAN and TRACTOR. The study design was different and health data were from different origin, with cancer registries for AGRICAN vs insurance health data for TRACTOR. AGRICAN did not consider farm managers and farm workers separately, focused on a limited part of France (11 counties/departments) and limited part of the agricultural population (7% of all active French agricultural workers covered by MSA). However, AGRICAN relied on high quality data (questionnaires) with more potential confounders and risk factors, with more accurate exposure ascertainment and the entire cursus laboris. Contrary to us, AGRICAN used a nonfarming population (eg, general population) as reference category. Despite these differences and limitations, the TRACTOR project yielded similar results than AGRICAN for many agricultural activities (Figure 1). Indeed, regardless of the CNS tumor type considered, similar findings than AGRICAN were found for several animal farming (both/mixed dairy and cow farming, ovine and caprine farming, stud farming, poultry farming) and viticulture. Regarding crop farming, an increased risk for CNS tumors and meningiomas were observed in both studies, but difference for gliomas were noticed (HR = 1.28 [1.01‐1.61] for TRACTOR vs 1.68 [0.88‐3.23] for AGRICAN). However, the comparison with AGRICAN results was not completely possible and optimal since the definition and description of activities were not the same (different activity coding system) in both studies.

#### 4.1.1 All CNS tumors

Increased risk of CNS cancers were found in mixed farming by five studies^7^, ^15^, ^16^, ^17^, ^18^ and in crop farming by two studies,^7^, ^19^ with results similar to ours. However, our risk estimations had narrower 95% CIs, likely due to a larger number of exposed cases. Regarding viticulture, two studies found an increased risk^11^, ^12^ while one found a decreased risk but for harvesting (OR = 0.62 [0.46‐0.82]).^10^ Our results are closer to the two studies that found an increased risk, with a positive trend observed in viticulture (HR = 1.21 [0.98‐1.48]). We found similar results than AGRICAN for crop farming (increased risk) and animal farming (Figure 1). The only difference was observed for pig farming for which we found an increased risk, in particular in men, while AGRICAN found a positive trend.

#### 4.1.2 Glioma

Eight studies have found an increased risk of glioma in overall agriculture.^2^ One French study found an increased risk in viticulture (OR = 3.21 [1.13‐9.11]).^11^ Another study reported a decreased risk in French viticulture for harvesting (OR = 0.50 [0.32‐0.76]) but an elevated risk for long pesticide exposures.^10^ Our results are closer to those of AGRICAN, with a positive trend observed in viticulture. Regarding crop farming, we found an increased risk while AGRICAN found a positive trend, possibly due to a larger number of exposed cases in TRACTOR (116 vs 79). Regarding animal farming, we found an increased risk in pig farming, in particular in men, while AGRICAN found no trend. We also observed differences of risk between women and men, in particular for dairy farming and for poultry and rabbit farming, suggesting potential gender specific tasks/exposures.

#### 4.1.3 Meningioma

One study reported a decreased risk of meningioma for open field farming in France (OR = 3.58 [1.20‐10.7]).^10^ Regarding crop farming, AGRICAN found a risk similar to ours (Figure 1). We found a positive trend for viticulture while AGRICAN reported no trend. Regarding animal farming, we found similar results than AGRICAN for most animal farming, with the exception of mixed dairy and cow farming for which we found an increased risk contrary to AGRICAN. This risk was higher for men than women, suggesting potential gender specific tasks/exposures. Regarding pig farming, AGRICAN reported an increased risk, which we did not find for overall meningioma, but that we observed for uncertain meningioma.

### 4.2 Risk factors

The etiology of CNS tumors is complex and involves many risk factors that could act differently according to subtype and that could play a role in the positive and negative associations that we found. Ionizing radiation exposure is the only established environmental risk factor for CNS tumors. Findings regarding other risk factors remain largely inconclusive.^2^, ^3^, ^4^, ^5^, ^20^, ^21^ Some intrinsic risk factors are suspected such as sex, ethnic group, genetic polymorphisms and syndromes, familial and personal predisposition and allergic conditions.^2^, ^3^, ^4^, ^21^ Several exogenous factors have also been proposed such as pesticide exposure, diet (nitroso compounds), hormones, smoking status, infection and seasonal effects, cell phone use, head trauma as well as reproductive factors.^3^, ^4^, ^5^, ^21^

Farmers are exposed to several physical, biological and chemical agents that can be potentially harmful. A recent review synthesizing 40 years of epidemiologic studies, including 20 cohorts, supports an increased risk of CNS cancer from farming related to potential pesticide exposure.^2^ Several studies found, for pesticide users and different pesticide classes, an increased risk of CNS tumors,^8^, ^9^, ^22^ gliomas^9^, ^23^ or meningioma.^8^, ^9^, ^24^ By contrast, one study found a decreased risk of gliomas for phenoxys exposure in the United States^25^ and a French case‐control study reported a decreased risk of gliomas and meningiomas for indirect pesticide exposures.^10^ We found no studies reporting either a decreased or an increased risk of CNS tumors other than gliomas or meningiomas related to pesticide exposures.

Biological risk factors are also of upmost interest. The use of pharmaceuticals in veterinary medicine, and in particular progestogens, could be a hypothesis to consider as they produce effects similar to those of the natural female sex hormone progesterone in the body and are, sometimes, associated with brain cancers, in particular meningiomas.^26^ In animal farming, progestogens are used to facilitate induction of normal estrous cycle activity in animals, in particular in swine/pig and horse breeding.^27^, ^28^ In addition, the role of infectious agents (eg, mycoplasma, viruses and bacteria) in the development of cancers, in particular for CNS tumors, have been considered recently.^29^, ^30^ For instance, some neurotropic viruses could lower^31^ or promote^32^ the risk of CNS tumors. While many farmers are exposed to vector‐borne diseases transmitted by animals or insects (eg, mosquitoes or ticks), the role of infectious agents in the occurrence of neoplasms remains controversial.

Although the etiology is unclear, there is suggestive evidence that parental occupational exposures could increase the risk of childhood brain tumors.^33^, ^34^, ^35^, ^36^, ^37^ Several studies reported positive associations between maternal prenatal occupational exposure to farm animals (pigs, horses, and poultry). By contrast, a pooled birth cohorts prospectively evaluating exposure to pesticides, animals, and organic dust in relation to childhood CNS tumor risk found no increased risks of CNS tumors related to paternal exposures to pesticides and animals using pooled data of 329 658 participants from birth cohorts in five countries (Australia, Denmark, Israel, Norway, and the United Kingdom).^38^

### 4.3 Strengths and limitations of this work

The most important strength of our study is the large number of exposed cases and completeness of available data. Because of the exhaustiveness of the population studied (entire French farm manager workforce) and because the reference group included only farm managers who did not carry out the activity of interest, the healthy worker effect remained limited. Compared to most studies, our study was restricted to farm managers. Farm managers and employees were not included in the same analysis due to different coding systems and data structure.^13^ In addition, farm managers and employees may have different socioeconomic status, different tasks and exposure, which could influence and bias risk estimation if not studied separately.^2^

CNS tumor cases were identified using ICD‐10 codes assigned to each worker that benefits from health care expenditure coverage for chronic diseases/long‐term illnesses, which is not comparable to the real illness incidences and could misestimate risk estimation. In particular, some benign tumors surgically removed early after diagnosis, preventing the need for health care expenditure coverage by MSA, might not be considered as long‐term illnesses. For this reason, the number of CNS cases, in particular meningiomas, was sometimes lower for TRACTOR compared to AGRICAN, which is based on cancer registries. The histological subtype was limited to 3‐digit long ICD‐10 codes, preventing the study of more descriptive subtype (eg, astrocytoma). However, subtypes that are more descriptive are rarely available in the literature and even if they were available, the number of exposed cases may not have been enough to conduct an analysis. A perspective for this work would be to confirm the cancer cases and their diagnosis with registry data from French departments with a cancer registry. However, it is currently impossible to link/pair individuals as they do not have the same unique anonymized identifier in the cancer registries and the TRACTOR project. Besides, linking cases with registry data requires proper authorizations from the independent administrative authority protecting privacy and personal data (CNIL) and cancer registries, which we do not have.

Another limitation pertained to the ascertainment of occupation and exposure. Only an indirect exposure estimation was possible using activities from administrative databases. An interesting future step will be to study the active ingredient utilized in agricultural activities using a crop‐exposure matrix such as Pestimat in order to ascertain more accurately the use of phytosanitary products.^39^ The downside of this approach is that information from crop‐exposure matrices are not available for each individual, but only at a large collective scale. Therefore, only a probability of pesticide use can be attributed to each farm manager based on available information (activity and location). In addition, the probability of pesticide used would be a rough estimation as the activities and locations available to TRACTOR are not descriptive enough to exploit the full potential of crop‐exposure matrices.

Although information on chemical, biological or physical agents encountered/used by farm managers and several potential confounders (eg, smoking and alcohol habits) were not available due to the inherent nature of available data (health insurance), risks were adjusted on important confounders (sex, age, geographical area) and on several covariates after a conservative selection based on the VIF (VIF ≤ 2.5). Confounding factors not available to the administrative health databases from TRACTOR and therefore not considered in this work could represent a bias. The potential impact of this bias on the results is hard to evaluate as these variables were not available. It is possible that their absence could bias the estimated effects and confounds/masks the genuine relationship between agricultural activities and CNS tumors. Findings should therefore be considered carefully. To refine analysis and address the aforementioned issue, external sources (eg, cohort studies and exposure matrices) could be linked to the TRACTOR project.^13^

In our study, age was considered in the models as a category rather than as a continuous variable. This choice was based on statistical consideration. Indeed, age did not follow a normal distribution and was moderately skewed to the right (data not shown). Categorizing continuous variables is a common practice in epidemiology.^40^ However, this practice has shortcomings such as loss of information, statistical power and increased probability of false negative findings (Type II error).^41^, ^42^ To reduce the loss of information and minimize the amount of residual confounding, we used four age categories.^43^ An alternative solution to the categorization of age could be to consider age as continuous in the models by using regression splines, smoothing splines or relax linearity with polynomial effects.^40^ However, these techniques also have limitations. For instance, determining the appropriate degree of smoothing to be applied is not straightforward as there is no widely accepted approach and may require expert knowledge and careful “tuning.”^44^, ^45^ To study the impact of categorizing age, we conducted a sensitivity analysis using age as a continuous variable in Cox model using the regression spline technique. In most cases (95.4%), results from the sensitivity analyses (Figures S6‐S8 and Tables S3 and S4 in the Supplemental Materials) yielded similar results than the approach using age as a categorical variable. Using age as continuous variable in the models tended to increase positive and negative findings. When using age as a category, there were a total of 40 and 2 activities that were found with increased and decreased risks of CNS tumors, respectively. By contrast, when using age as a continuous variable, there were a total of 43 and 6 activities that were found with increased and decreased risks of CNS tumors, respectively. Cattle farming activities (dairy and cow farming) were the only agricultural activities that differed in the observation of decreased risk of CNS tumors between both analyses. These activities were more often found with decreased risks (6 vs 2) with the sensitivity analysis than with the analysis using age as a category. However, estimated risks were very similar for both analyses. For instance, for dairy farming performed by women, risk of CNS tumors (0.77 [0.57‐1.03] vs 0.73 [0.55‐0.97]), glioma (0.57 [0.33‐0.98] vs 0.60 [0.35‐1.03]) and benign meningioma (0.52 [0.26‐1.04] vs 0.49 [0.25‐0.97]) were comparable. Regarding agricultural activities that differed in the observation of increased risk between both analyses, there were 27 noticeable differences. Most of these differences were observed for crop farming (33%), unspecified and mixed farming (15%), fruit arboriculture (7%) and truck farming, floriculture/flower‐growing (7%). For crop farming, the sensitivity analysis yielded 9 more increased risks of CNS tumors, which were 1.2 to 2.1 times higher than the results from the analysis using age as a categorical variable. Regarding truck farming, floriculture/flower‐growing, risks were found to be 1.1 to 1.3 times higher with the sensitivity analysis, while for fruit arboriculture, 2 models from the sensitivity analysis yielded risks 1.1 times lower than when using age as a category.

There were a few activities that were found with decreased risks of CNS tumors compared to activities that were found with increased risks. This may be explained by the fact that “potential confounders” differed from a model to another due to the variable selection process (based on the VIF) and because the reference group differed from an activity to another.

To lessen the possibility of chance findings, we conducted an analysis only when the number of exposed cases was ≥3. False associations resulting from multiple comparisons might be an issue in our analysis, but approaches used to limit false positive findings (Type I errors) (eg, Benjamini‐Hochberg procedure) are too conservative, increase the risk of false negative findings (Type II errors) and are not relevant in the framework of large cohort study with data on multiple illnesses.^46^

In our study, we chose the time to first CNS tumor insurance declaration as the underlying timescale. The choice of the time scale is highly discussed in the literature but, to the best of our knowledge, there is no general consensus on which time scale is the most appropriate for a given question or study. According to several studies, using time‐on‐study models may be preferable since these models perform at least as well as the left truncated age scale model, and also because they are more robust to misspecification of the underlying time scale and have better predictive ability in general.^47^, ^48^, ^49^ There has been some differences in the associations found in this work and the ones from literature. Some differences may be explained by the difference in the study design, health data origin and by different temporal and geographical scales. However, cohort studies adjust on more potential confounders and rely usually on more accurate/descriptive exposure ascertainment. Despite these differences, many findings were consistent with existing literature, but with more exposed cases, narrower 95% CIs and information on both sexes and several CNS tumor types that have been rarely studied before. Nevertheless, findings should be considered carefully by taking into account, the number, the direction and the magnitude of all examined risk associations.

In conclusion, the TRACTOR project brings new insights and a wealth of information on the association of a wide range of agricultural activities and CNS tumor and type‐specific risks in farm managers, overall and for both sexes. The completeness of data and the large number of exposed cases offered a unique opportunity to study a rare disease such as CNS tumor. Results from our study are complementary to cohort studies and allow the identification of agricultural activities at risk where further studies are needed, which could have broad implications for disease surveillance in agriculture.

## AUTHOR CONTRIBUTIONS

**Pascal Petit**: Conceptualization, Methodology, Software, Validation, Formal analysis, Investigation, Data Curation, Writing—Original Draft, Writing—Review & Editing, Visualization. **Gérald Gandon**: Writing—Review & Editing. **Stéphan Chabardes**: Writing—Review & Editing. **Vincent Bonneterre**: Writing—Review & Editing. The work reported in the paper has been performed by the authors, unless clearly specified in the text.

## FUNDING INFORMATION

This work has been partially supported by MIAI@Grenoble Alpes (ANR‐19‐P3IA‐0003, 2019) and by the French Agency for Food, Environmental and Occupational Health & Safety (2016‐CRD‐03_PPV16/534B, 2016; 2018‐CRD‐14_PPV18, 2018). The funding sources had no role in the study design; in the collection, analysis, and interpretation of data; in the writing of the report; or in the decision to submit the paper for publication. The authors declare no conflict of interest relating to the material presented in this article. Its contents, including any opinions and/or conclusions expressed, are solely those of the authors.

## CONFLICT OF INTEREST

The authors declare that they have no known competing financial interests or personal relationships that could have appeared to influence the work reported in this paper.

## ETHICS STATEMENT

The use of MSA data for the TRACTOR project was approved by the French independent administrative authority protecting privacy and personal data (CNIL) (authorization number MMS/SBM/AE171001). Following CNIL instructions, MSA is required to make bill posting in each of its 35 offices and to communicate yearly to all of its insured individuals about the goals, advancements and achievements of the TRACTOR project. No informed consent was required by CNIL for the TRACTOR project because data analyses were only descriptive and results were reported at a large collective scale (ie, activity level), because data were pseudomyzed and because measures were undertaken to prevent the risk of reidentification of individuals.

## Supporting information


**Appendix S1** Supporting Information

## Data Availability

The data that support the findings of our study are available upon reasonable request to the Mutualité Sociale Agricole (MSA) but restrictions apply to the availability of these data, which were used under MSA approval and were approved by the French independent administrative authority protecting privacy and personal data (CNIL) for the current study, and so are not publicly available. Further information is available from the corresponding author upon request.
