# Supplementary material for: Agricultural activities and risk of central nervous system tumors among French farm managers: Results from the TRACTOR project
Source: Int J Cancer. 2022 Jul 16;151(10):1737–49. doi: 10.1002/ijc.34197 (PMC9796624; doi:10.1002/ijc.34197)
Supplement: Supplementary file 1 — Appendix S1 Supporting Information [file IJC-151-1737-s001.pdf]

Supplementary material

Title: Agricultural activities and risk of central nervous system tumors among French farm managers: results from the TRACTOR project

Authors

Pascal Petit[1,*], Gérald Gandon[2] , Stéphan Chabardès[3], Vincent Bonneterre[1]

Table of contents

Table S1: Agricultural practices and risks of CNS tumors and meningiomas, TRACTOR, France, 2002-2016 2

Table S2: Agricultural practices and risks of gliomas and tumors of spinal cord, cranial nerves and other parts of CNS, TRACTOR, France, 2002-2016 4

Table S3: Results from sensitivity analysis (age as continuous variable) - Agricultural practices and risks of CNS tumors and meningiomas, TRACTOR, France, 2002-2016 6

Table S4: Results from sensitivity analysis (age as continuous variable) - Agricultural practices and risks of gliomas and tumors of spinal cord, cranial nerves and other parts of CNS, TRACTOR, France, 2002-2016........ 8

Fig. S1: Number of farm managers by agricultural activities and sex 10

Fig. S2: Percentage of CNS tumors by sex, activity and CNS type........ 11

Fig. S3: Agricultural practices and risks of CNS tumors, TRACTOR, France, 2002-2016 12

Fig.S4: Agricultural practices and risks of CNS tumors for women, TRACTOR, France, 2002-2016 13

Fig.S5: Agricultural practices and risks of CNS tumors for men, TRACTOR, France, 2002-2016..... 14

Fig.S6: Results from sensitivity analysis (age as continuous variable) - Agricultural practices and risks of CNS tumors for both sexes combined, TRACTOR, France, 2002-2016 15

Fig. S7: Results from sensitivity analysis (age as continuous variable) - Agricultural practices and risks of CNS tumors for women, TRACTOR, France, 2002-2016........ 16

Fig. S8: Results from sensitivity analysis (age as continuous variable) - Agricultural practices and risks of CNS tumors for men, TRACTOR, France, 2002-2016........ 17

Table S1: Agricultural practices and risks of CNS tumors and meningiomas, TRACTOR, France, 2002-2016

Agricultural practice/activity	Sex	Study population	All CNS tumors		All meningiomas		C70		D32		D42	
		(%)	m[a] (%)	HR[b] [95% CI]	m[c] (%)	HR[b] [95% CI]	m[c] (%)	HR[b] [95% CI]	m[c] (%)	HR[b] [95% CI]	m[c] (%)	HR[b] [95% CI]
Both/mixed dairy and cow farming	Both sexes	30,729 (3.0)	36 (3.5)	0.98 [0.70 - 1.37]	19 (52.7)	**1.75 [1.09 - 2.81]**	5 (13.9)	1.62 [0.64 - 4.09]	5 (13.9)	0.91 [0.37 - 2.24]	9 (25.0)	**2.67 [1.32 - 5.39]**
(individuals performing both dairy and cow farming)	Woman	8,004 (2.5)	15 (3.8)	1.13 [0.67 - 1.91]	7 (46.7)	1.06 [0.49 - 2.28]	2 (13.3)	NC	2 (13.3)	NC	3 (20.0)	1.38 [0.42 - 4.50]
	Man	22,725 (3.2)	21 (3.4)	0.89 [0.57 - 1.39]	12 (57.1)	**2.69 [1.46 - 4.97]**	3 (14.3)	2.14 [0.63 - 7.26]	3 (14.3)	1.33 [0.41 - 4.36]	6 (28.6)	**4.83 [1.95 - 12.0]**
Cow farming	Both sexes	110,214 (10.6)	121 (11.9)	1.03 [0.85 - 1.25]	46 (38.0)	1.30 [0.94 - 1.79]	9 (7.4)	1.05 [0.51 - 2.14]	29 (24.0)	**1.69 [1.11 - 2.56]**	9 (7.4)	0.72 [0.36 - 1.45]
(individuals performing only cow farming)	Woman	32,699 (10.2)	50 (12.5)	1.11 [0.82 - 1.50]	30 (60.0)	1.34 [0.90 - 2.00]	5 (10.0)	1.11 [0.43 - 2.89]	20 (40.0)	**1.68 [1.01 - 2.79]**	6 (12.0)	0.83 [0.35 - 1.97]
	Man	77,515 (10.8)	71 (11.5)	0.97 [0.75 - 1.25]	16 (22.5)	1.17 [0.68 - 2.01]	4 (5.6)	1.12 [0.38 - 3.31]	9 (12.7)	1.58 [0.75 - 3.31]	3 (4.2)	0.51 [0.16 - 1.66]
Dairy farming	Both sexes	158,706 (15.3)	186 (18.3)	1.04 [0.87 - 1.23]	45 (24.2)	0.78 [0.56 - 1.10]	18 (9.7)	1.25 [0.69 - 2.24]	20 (10.8)	0.74 [0.45 - 1.21]	12 (6.5)	0.62 [0.33 - 1.18]
(individuals performing only dairy farming)	Woman	48,823 (15.2)	62 (15.5)	0.77 [0.57 - 1.03]	26 (41.9)	0.69 [0.44 - 1.07]	10 (16.1)	1.29 [0.58 - 2.89]	10 (16.1)	0.52 [0.26 - 1.04]	10 (16.1)	0.79 [0.38 - 1.67]
	Man	109,883 (15.4)	124 (20.0)	1.22 [0.98 - 1.52]	19 (15.3)	0.80 [0.48 - 1.36]	8 (6.5)	1.22 [0.51 - 2.95]	10 (8.1)	1.00 [0.48 - 2.09]	2 (1.6)	NC
Ovine and caprine farming	Both sexes	47,086 (4.5)	45 (4.4)	1.17 [0.86 - 1.58]	16 (35.6)	1.47 [0.88 - 2.45]	2 (4.4)	NC	6 (13.3)	1.10 [0.48 - 2.52]	8 (17.8)	**2.27 [1.08 - 4.76]**
	Woman	16,808 (5.2)	16 (4.0)	0.86 [0.52 - 1.44]	9 (56.3)	0.99 [0.50 - 1.96]	1 (6.3)	NC	4 (25.0)	0.76 [0.27 - 2.08]	4 (25.0)	1.61 [0.57 - 4.57]
	Man	30,278 (4.2)	29 (4.7)	1.33 [0.91 - 1.94]	7 (24.1)	1.95 [0.89 - 4.25]	1 (3.4)	NC	2 (6.7)	NC	4 (4.7)	2.50 [0.87 - 7.20]
Pig farming	Both sexes	13,389 (1.3)	24 (2.4)	**1.67 [1.10 - 2.54]**	6 (25.0)	1.58 [0.68 - 3.64]	1 (4.2)	NC	1 (4.2)	NC	4 (16.7)	**3.18 [1.11 - 9.16]**
	Woman	3,830 (1.2)	5 (1.3)	0.95 [0.38 - 2.33]	2 (40.0)	NC	0	NC	1 (20.0)	NC	1 (20.0)	NC
	Man	9,559 (1.3)	19 (3.1)	**2.18 [1.35 - 3.52]**	4 (21.0)	2.53 [0.88 - 7.24]	1 (5.3)	NC	0	NC	3 (15.8)	**8.11 [2.29 - 28.7]**
Poultry and rabbit farming	Both sexes	24,576 (2.4)	25 (2.5)	1.13 [0.76 - 1.69]	6 (24.0)	0.93 [0.41 - 2.09]	1 (4.0)	NC	2 (8.0)	NC	3 (12.0)	1.36 [0.42 - 4.34]
	Woman	9,671 (3.0)	14 (3.5)	1.22 [0.71 - 2.11]	4 (28.6)	0.70 [0.25 - 1.91]	1 (7.1)	NC	1 (7.1)	NC	2 (14.3)	NC
	Man	14,905 (2.1)	11 (1.8)	0.92 [0.51 - 1.68]	2 (18.2)	NC	0	NC	1 (9.1)	NC	1 (9.1)	NC
Stud farming	Both sexes	15,641 (1.5)	6 (0.6)	0.85 [0.38 - 1.91]	3 (50.0)	1.53 [0.49 - 4.81]	0	NC	3 (50.0)	3.16 [0.99 - 10.0]	0	NC
	Woman	6,831 (2.1)	5 (1.3)	1.14 [0.47 - 2.82]	2 (40.0)	NC	0	NC	2 (40.0)	NC	0	NC
	Man	8,810 (1.2)	1 (0.2)	NC	1 (100)	NC	0	NC	1 (100)	NC	0	NC
Training, dressage, riding clubs	Both sexes	13,273 (1.3)	8 (0.8)	0.91 [0.45 - 1.83]	2 (25.0)	NC	1 (12.5)	NC	0	NC	1 (12.5)	NC
	Woman	6,049 (1.9)	2 (0.5)	NC	1 (50.0)	NC	1 (50.0)	NC	0	NC	0	NC
	Man	7,224 (1.0)	6 (1.0)	1.34 [0.60 - 3.02]	1 (16.7)	NC	0	NC	0	NC	1 (16.7)	NC
Unspecified large animal farming	Both sexes	2,663 (0.3)	4 (0.4)	**3.67 [1.37 - 9.82]**	2 (50.0)	NC	1 (25.0)	NC	1 (25.0)	NC	0	NC
(e.g. large dogs, zoo)	Woman	1,280 (0.4)	3 (0.8)	**5.71 [1.82 - 17.9]**	2 (66.7)	NC	1 (33.3)	NC	1 (33.3)	NC	0	NC
	Man	1,383 (0.2)	1 (0.2)	NC	0	NC	0	NC	0	NC	0	NC
Unspecified small animal farming	Both sexes	18,058 (1.7)	10 (1.0)	1.56 [0.84 - 2.91]	3 (30.0)	1.54 [0.49 - 4.80]	0	NC	2 (20.0)	NC	1 (10.0)	NC
(e.g. frogs, snails, bees)	Woman	7,698 (2.4)	5 (1.3)	1.88 [0.77 - 4.59]	1 (20.0)	NC	0	NC	2 (40.0)	NC	0	NC
	Man	10,360 (1.4)	5 (0.8)	1.28 [0.53 - 3.09]	1 (20.0)	NC	0	NC	0	NC	1 (20.0)	NC
Fruit arboriculture	Both sexes	24,086 (2.3)	27 (2.7)	**1.50 [1.02 - 2.21]**	3 (11.1)	0.5 [0.16 - 1.56]	0	NC	2 (7.4)	NC	1 (3.7)	NC
	Woman	7,649 (2.4)	10 (2.5)	1.25 [0.66 - 2.35]	2 (20.0)	NC	0	NC	1 (10.0)	NC	1 (10.0)	NC
	Man	16,437 (2.3)	17 (1.1)	**1.67 [1.03 - 2.72]**	1 (5.9)	NC	0	NC	1 (5.9)	NC	0	NC
Garden center	Both sexes	5,111 (0.5)	3 (0.3)	0.87 [0.28 - 2.70]	2 (66.7)	NC	0	NC	2 (66.7)	NC	0	NC
	Woman	1,358 (0.4)	2 (0.5)	NC	2 (100)	NC	0	NC	2 (100)	NC	0	NC
	Man	3,753 (0.5)	1 (0.2)	NC	0	NC	0	NC	0	NC	0	NC
Truck farming, floriculture/flower-growing	Both sexes	41,525 (4.0)	44 (4.3)	**1.36 [1.00 - 1.84]**	14 (31.8)	1.46 [0.85 - 2.51]	3 (6.8)	1.79 [0.56 - 5.73]	8 (18.2)	1.69 [0.82 - 3.48]	3 (6.8)	0.87 [0.27 - 2.76]
	Woman	12,672 (4.0)	17 (4.3)	1.40 [0.86 - 2.29]	8 (47.0)	1.45 [0.71 - 2.97]	2 (11.8)	NC	4 (23.5)	1.32 [0.48 - 3.64]	2 (11.8)	NC
	Man	28,853 (4.0)	27 (4.4)	1.36 [0.92 - 2.01]	6 (22.2)	1.58 [0.69 - 3.62]	1 (3.7)	NC	4 (14.8)	2.40 [0.85 - 6.77]	1 (3.7)	NC
Unspecified and mixed farming	Both sexes	120,746 (11.7)	140 (13.8)	**1.34 [1.12 - 1.61]**	36 (25.7)	1.10 [0.77 - 1.56]	7 (5.0)	0.88 [0.40 - 1.94]	12 (8.6)	0.72 [0.40 - 1.31]	20 (14.3)	**1.83 [1.11 - 3.03]**
(e.g. polyculture, mixed farming)	Woman	36,955 (11.5)	59 (14.8)	**1.54 [1.16 - 2.05]**	25 (42.4)	1.28 [0.83 - 1.96]	4 (6.8)	1.11 [0.39 - 3.16]	9 (15.3)	0.82 [0.41 - 1.63]	14 (23.7)	**2.34 [1.26 - 4.35]**
	Man	83,791 (11.7)	81 (13.1)	1.21 [0.96 - 1.54]	11 (13.6)	0.77 [0.41 - 1.44]	3 (3.7)	0.84 [0.25 - 2.83]	3 (3.7)	0.47 [0.15 - 1.53]	6 (7.4)	1.09 [0.45 - 2.64]
Unspecified specialized crop farming	Both sexes	6,168 (0.6)	4 (0.4)	1.21 [0.45 - 3.23]	2 (50.0)	NC	0	NC	2 (50.0)	NC	0	NC
(e.g. horticulture)	Woman	2,231 (0.7)	2 (0.5)	NC	1 (50.0)	NC	0	NC	1 (50.0)	NC	0	NC
	Man	3,933 (0.5)	2 (0.3)	NC	1 (50.0)	NC	0	NC	1 (50.0)	NC	0	NC
Viticulture	Both sexes	118,577 (11.4)	113 (11.1)	1.21 [0.98 - 1.48]	40 (35.4)	1.31 [0.92 - 1.85]	6 (5.3)	0.97 [0.41 - 2.33]	22 (19.5)	1.59 [0.99 - 2.55]	13 (11.5)	1.15 [0.62 - 2.11]
	Woman	41,970 (13.1)	49 (12.3)	1.29 [0.94 - 1.77]	27 (55.1)	1.48 [0.96 - 2.28]	6 (12.2)	2.12 [0.83 - 5.43]	15 (30.6)	1.54 [0.86 - 2.75]	7 (14.3)	1.02 [0.44 - 2.36]
	Man	76,607 (10.7)	64 (10.4)	1.14 [0.87 - 1.49]	13 (20.3)	1.08 [0.60 - 1.97]	0	NC	7 (10.9)	1.76 [0.76 - 4.04]	6 (9.4)	1.19 [0.48 - 2.97]
Crop farming (including field crops, cereal grain crops,	Both sexes	305,838 (29.5)	258 (25.4)	**1.20 [1.03 - 1.41]**	87 (33.7)	1.13 [0.86 - 1.48]	19 (7.4)	1.43 [0.80 - 2.55]	46 (17.8)	1.18 [0.81 - 1.73]	29 (11.2)	1.05 [0.66 - 1.67]
wheat and industrial grower)	Woman	102,240 (31.9)	113 (28.3)	1.11 [0.87 - 1.41]	55 (48.7)	1.17 [0.82 - 1.66]	11 (9.7)	1.04 [0.48 - 2.27]	32 (28.3)	1.24 [0.77 - 2.00]	17 (15.0)	1.13 [0.60 - 2.12]
	Man	203,598 (28.4)	145 (23.5)	1.21 [0.99 - 1.47]	32 (22.1)	1.18 [0.77 - 1.83]	8 (5.5)	1.80 [0.75 - 4.36]	14 (9.7)	1.21 [0.62 - 2.36]	12 (8.3)	1.03 [0.51 - 2.10]

Agricultural work companies	Both sexes	14,282 (1.4)	11 (1.1)	1.04 [0.58 - 1.90]	4 (36.4)	1.35 [0.50 - 3.62]	0	NC	1 (9.1)	NC	3 (27.3)	3.05 [0.96 - 9.68]
	Woman	1,715 (0.5)	0	NC	0	NC	0	NC	0	NC	0	NC
	Man	12,567 (1.8)	11 (1.8)	1.41 [0.77 - 2.56]	4 (36.4)	2.69 [0.99 - 7.35]	0	NC	1 (9.1)	NC	3 (27.3)	**5.46 [1.66 - 17.9]**
Company representative/authorized representative	Both sexes	1,846 (0.2)	3 (0.3)	**4.15 [1.33 - 12.9]**	1 (33.3)	NC	0	NC	1 (33.3)	NC	0	NC
	Woman	1,435 (0.4)	2 (0.5)	NC	1 (50.0)	NC	0	NC	1 (50.0)	NC	0	NC
	Man	408 (0.06)	1 (0.2)	NC	0	NC	0	NC	0	NC	0	NC
Gardening, landscaping and reforestation companies	Both sexes	44,948 (4.3)	28 (2.8)	0.87 [0.59 - 1.28]	5 (17.9)	0.59 [0.24 - 1.45]	3 (10.7)	2.23 [0.68 - 7.26]	2 (7.1)	NC	0	NC
	Woman	2,369 (0.7)	7 (1.8)	**6.25 [2.91 - 13.4]**	3 (42.9)	**5.94 [1.85 - 19.0]**	2 (28.6)	NC	1 (14.3)	NC	0	NC
	Man	42,579 (5.9)	21 (3.4)	0.86 [0.55 - 1.34]	2 (9.5)	NC	1 (4.8)	NC	1 (4.8)	NC	0	NC
Wood production	Both sexes	10,470 (1.0)	10 (1.0)	0.83 [0.44 - 1.55]	2 (20.0)	NC	0	NC	1 (10.0)	NC	1 (10.0)	NC
	Woman	283 (0.09)	0	NC	0	NC	0	NC	0	NC	0	NC
	Man	10,187 (1.4)	10 (1.6)	1.07 [0.57 - 2.01]	2 (20.0)	NC	0	NC	1 (10.0)	NC	1 (10.0)	NC
Shellfish farming	Both sexes	3,350 (0.3)	2 (0.2)	NC	0	NC	0	NC	0	NC	0	NC
	Woman	666 (0.2)	0	NC	0	NC	0	NC	0	NC	0	NC
	Man	2,684 (0.4)	2 (0.3)	NC	0	NC	0	NC	0	NC	0	NC
Salt marsh	Both sexes	873 (0.08)	0	NC	0	NC	0	NC	0	NC	0	NC
	Woman	200 (0.06)	0	NC	0	NC	0	NC	0	NC	0	NC
	Man	673 (0.09)	0	NC	0	NC	0	NC	0	NC	0	NC
Fixed sawmill	Both sexes	735 (0.07)	0	NC	0	NC	0	NC	0	NC	0	NC
	Woman	48 (0.01)	0	NC	0	NC	0	NC	0	NC	0	NC
	Man	687 (0.1)	0	NC	0	NC	0	NC	0	NC	0	NC
Rural craftsperson	Both sexes	7,038 (0.7)	0	NC	0	NC	0	NC	0	NC	0	NC
	Woman	256 (0.08)	0	NC	0	NC	0	NC	0	NC	0	NC
	Man	6,782 (0.9)	0	NC	0	NC	0	NC	0	NC	0	NC
Sylviculture/forestry	Both sexes	1,986 (0.2)	2 (0.2)	NC	0	NC	0	NC	0	NC	0	NC
	Woman	339 (0.1)	0	NC	0	NC	0	NC	0	NC	0	NC
	Man	1,647 (0.2)	2 (0.3)	NC	0	NC	0	NC	0	NC	0	NC

Note: m: number of exposed cases, NC: not calculated, CNS: central nervous system, C70: malignant neoplasm of meninges, D32: benign neoplasm of meninges, D42: neoplasm of uncertain or unknown behavior of meninges, 95% CI: 95% confidence interval.

[a] The percentages in brackets refer to the ratio of exposed cases in the study population and the total number of cases in the overall population.

[b] Hazard ratios were estimated by Cox models with time to first CNS tumor insurance declaration as the underlying timescale, when the number of exposed cases was sufficient ($m \geq 3$), adjusted for sex (for "both sexes" only), age, first year of the farm's establishment, farm surface, earnings, number of associates, unemployment status, total number of farms, family status, partner work status, farm location, number of comorbidities and having a secondary activity.

[c] The percentages in brackets refer to the ratio of exposed cases in the study population and the total number of cases in the study population.

Table S2: Agricultural practices and risks of gliomas and tumors of spinal cord, cranial nerves and other parts of CNS, TRACTOR, France, 2002-2016

Agricultural practice/activity	Sex	Study population	C71		D33		D43		C72	
		(%)	m[c] (%)	HR[b] [95% CI]	m[c] (%)	HR[b] [95% CI]	m[c] (%)	HR[b] [95% CI]	m[c] (%)	HR[b] [95% CI]
Both/mixed dairy and cow farming (individuals performing both dairy and cow farming)	Both sexes	30,729 (3.0)	11 (30.6)	0.62 [0.34 - 1.13]	1 (2.8)	NC	5 (13.9)	1.24 [0.50 - 3.10]	0	NC
	Woman	8,004 (2.5)	5 (33.3)	1.01 [0.41 - 2.49]	0	NC	3 (20.0)	2.81 [0.83 - 9.51]	0	NC
	Man	22,725 (3.2)	6 (28.6)	**0.43 [0.19 - 0.97]**	1 (4.8)	NC	2 (9.5)	NC	0	NC
Cow farming (individuals performing only cow farming)	Both sexes	110,214 (10.6)	45 (37.2)	0.78 [0.57 - 1.07]	7 (5.8)	0.63 [0.29 - 1.39]	21 (17.4)	**1.84 [1.11 - 3.02]**	2 (1.7)	NC
	Woman	32,699 (10.2)	10 (20.0)	0.59 [0.31 - 1.14]	3 (6.0)	0.78 [0.23 - 2.61]	7 (14.0)	2.25 [0.96 - 5.28]	0	NC
	Man	77,515 (10.8)	35 (49.3)	0.82 [0.57 - 1.17]	4 (5.6)	0.56 [0.20 - 1.58]	14 (19.7)	1.78 [0.96 - 3.28]	2 (2.8)	NC
Dairy farming (individuals performing only dairy farming)	Both sexes	158,706 (15.3)	99 (53.2)	1.18 [0.92 - 1.51]	16 (8.6)	1.53 [0.84 - 2.81]	16 (8.6)	0.77 [0.43 - 1.37]	13 (7.0)	**2.10 [1.01 - 4.34]**
	Woman	48,823 (15.2)	18 (29.0)	**0.57 [0.33 - 0.98]**	8 (12.9)	2.01 [0.81 - 4.97]	6 (9.7)	0.66 [0.25 - 1.72]	6 (9.7)	**2.77 [1.04 - 7.38]**
	Man	109,883 (15.4)	81 (65.3)	**1.43 [1.08 - 1.89]**	8 (6.5)	1.38 [0.61 - 3.13]	10 (8.1)	0.83 [0.40 - 1.72]	7 (5.6)	1.67 [0.63 - 4.43]
Ovine and caprine farming	Both sexes	47,086 (4.5)	23 (51.1)	1.26 [0.82 - 1.93]	4 (8.9)	1.23 [0.44 - 3.40]	4 (8.9)	0.77 [0.28 - 2.14]	0	NC
	Woman	16,808 (5.2)	4 (25.0)	0.63 [0.23 - 1.72]	3 (18.8)	2.28 [0.67 - 7.74]	1 (6.3)	NC	0	NC
	Man	30,278 (4.2)	19 (65.5)	1.55 [0.96 - 2.49]	1 (3.4)	NC	3 (10.3)	0.89 [0.27 - 2.91]	0	NC
Pig farming	Both sexes	13,389 (1.3)	17 (70.8)	**2.28 [1.37 - 3.80]**	0	NC	0	NC	1 (4.2)	NC
	Woman	3,830 (1.2)	3 (60.0)	1.46 [0.45 - 4.74]	0	NC	0	NC	0	NC
	Man	9,559 (1.3)	14 (73.7)	**2.52 [1.43 - 4.45]**	0	NC	0	NC	1 (5.3)	NC
Poultry and rabbit farming	Both sexes	24,576 (2.4)	13 (52.0)	1.14 [0.65 - 2.00]	3 (12.0)	1.72 [0.53 - 5.55]	2 (8.0)	NC	2 (8.0)	NC
	Woman	9,671 (3.0)	8 (57.1)	**2.31 [1.10 - 4.85]**	1 (7.1)	NC	0	NC	2 (14.3)	NC
	Man	14,905 (2.1)	5 (45.5)	0.66 [0.27 - 1.61]	2 (18.2)	NC	2 (18.2)	NC	0	NC
Stud farming	Both sexes	15,641 (1.5)	3 (50.0)	0.84 [0.27 - 2.62]	0	NC	0	NC	0	NC
	Woman	6,831 (2.1)	3 (60.0)	1.99 [0.61 - 6.52]	0	NC	0	NC	0	NC
	Man	8,810 (1.2)	0	NC	0	NC	0	NC	0	NC
Training, dressage, riding clubs	Both sexes	13,273 (1.3)	4 (50.0)	0.83 [0.31 - 2.24]	0	NC	2 (25.0)	NC	0	NC
	Woman	6,049 (1.9)	1 (50.0)	NC	0	NC	0	NC	0	NC
	Man	7,224 (1.0)	3 (50.0)	1.16 [0.37 - 3.64]	0	NC	2 (33.3)	NC	0	NC
Unspecified large animal farming (e.g. large dogs, zoo)	Both sexes	2,663 (0.3)	1 (25.0)	NC	0	NC	1 (25.0)	NC	0	NC
	Woman	1,280 (0.4)	0	NC	0	NC	1 (33.3)	NC	0	NC
	Man	1,383 (0.2)	1 (100)	NC	0	NC	0	NC	0	NC
Unspecified small animal farming (e.g. frogs, snails, bees)	Both sexes	18,058 (1.7)	4 (40.0)	1.31 [0.49 - 3.52]	1 (10.0)	NC	2 (20.0)	NC	0	NC
	Woman	7,698 (2.4)	1 (20.0)	NC	1 (20.0)	NC	1 (20.0)	NC	0	NC
	Man	10,360 (1.4)	3 (60.0)	1.37 [0.44 - 4.28]	0	NC	1 (20.0)	NC	0	NC
Fruit arboriculture	Both sexes	24,086 (2.3)	14 (51.9)	**1.72 [1.00 - 2.94]**	3 (11.1)	2.11 [0.66 - 6.79]	6 (22.2)	**3.05 [1.31 - 7.08]**	2 (7.4)	NC
	Woman	7,649 (2.4)	6 (60.0)	**2.58 [1.12 - 5.96]**	1 (10.0)	NC	1 (10.0)	NC	0	NC
	Man	16,437 (2.3)	8 (47.1)	1.36 [0.67 - 2.76]	2 (11.8)	NC	5 (29.4)	**4.02 [1.57 - 10.3]**	2 (11.8)	NC
Garden center	Both sexes	5,111 (0.5)	0	NC	1 (33.3)	NC	0	NC	0	NC
	Woman	1,358 (0.4)	0	NC	0	NC	0	NC	0	NC
	Man	3,753 (0.5)	0	NC	1 (100)	NC	0	NC	0	NC
Truck farming, floriculture/flower-growing	Both sexes	41,525 (4.0)	17 (38.6)	1.08 [0.66 - 1.76]	3 (6.8)	1.11 [0.35 - 3.56]	6 (13.6)	1.52 [0.66 - 3.51]	4 (9.1)	**3.63 [1.27 - 10.4]**
	Woman	12,672 (4.0)	5 (29.4)	1.23 [0.50 - 3.03]	1 (5.9)	NC	2 (11.8)	NC	1 (5.9)	NC
	Man	28,853 (4.0)	12 (44.4)	1.02 [0.57 - 1.82]	2 (7.4)	NC	4 (14.8)	1.58 [0.57 - 4.38]	3 (11.1)	**5.42 [1.55 - 18.9]**
Unspecified and mixed farming (e.g. polyculture, mixed farming)	Both sexes	120,746 (11.7)	72 (51.4)	**1.52 [1.17 - 1.96]**	13 (9.3)	1.47 [0.80 - 2.70]	17 (12.1)	1.48 [0.87 - 2.52]	6 (4.3)	1.04 [0.43 - 2.53]
	Woman	36,955 (11.5)	19 (32.2)	1.34 [0.81 - 2.21]	5 (8.5)	1.94 [0.73 - 5.15]	7 (11.9)	2.27 [0.98 - 5.28]	5 (8.5)	2.23 [0.79 - 6.26]
	Man	83,791 (11.7)	53 (65.4)	**1.47 [1.09 - 1.98]**	8 (9.9)	1.24 [0.57 - 2.68]	10 (12.3)	1.23 [0.62 - 2.43]	1 (1.2)	NC
Unspecified specialized crop farming (e.g. horticulture)	Both sexes	6,168 (0.6)	1 (25.0)	NC	1 (25.0)	NC	0	NC	0	NC
	Woman	2,231 (0.7)	1 (50.0)	NC	0	NC	0	NC	0	NC
	Man	3,933 (0.5)	0	NC	1 (50.0)	NC	0	NC	0	NC
Viticulture	Both sexes	118,577 (11.4)	53 (46.9)	1.29 [0.95 - 1.74]	12 (10.6)	1.49 [0.78 - 2.84]	8 (7.1)	0.70 [0.33 - 1.47]	1 (0.9)	NC
	Woman	41,970 (13.1)	17 (34.7)	1.57 [0.91 - 2.71]	3 (6.1)	0.83 [0.24 - 2.87]	2 (4.1)	NC	0	NC
	Man	76,607 (10.7)	36 (56.3)	1.16 [0.81 - 1.67]	9 (14.1)	2.00 [0.94 - 4.29]	6 (9.4)	0.87 [0.37 - 2.06]	1 (1.6)	NC
Crop farming (including field crops, cereal grain crops, wheat and industrial grower)	Both sexes	305,838 (29.5)	116 (45.0)	**1.28 [1.01 - 1.61]**	29 (11.2)	**1.64 [1.00 - 2.69]**	21 (8.1)	1.04 [0.62 - 1.76]	11 (4.3)	1.66 [0.77 - 3.59]
	Woman	102,240 (31.9)	36 (31.9)	1.12 [0.72 - 1.74]	11 (9.7)	1.19 [0.53 - 2.64]	10 (8.8)	0.99 [0.44 - 2.22]	4 (3.5)	1.10 [0.36 - 3.35]
	Man	203,598 (28.4)	80 (55.2)	1.22 [0.93 - 1.59]	18 (12.4)	**1.88 [1.01 - 3.50]**	11 (7.6)	0.76 [0.38 - 1.53]	7 (4.8)	2.32 [0.90 - 5.95]

Agricultural work companies	Both sexes	14,282 (1.4)	4 (36.4)	0.77 [0.29 - 2.07]	2 (18.2)	NC	1 (9.1)	NC	0	NC
	Woman	1,715 (0.5)	0	NC	0	NC	0	NC	0	NC
	Man	12,567 (1.8)	4 (36.4)	0.91 [0.34 - 2.45]	2 (18.2)	NC	1 (9.1)	NC	0	NC
Company representative/authorized representative	Both sexes	1,846 (0.2)	1 (33.3)	NC	0	NC	1 (33.3)	NC	0	NC
	Woman	1,435 (0.4)	0	NC	0	NC	1 (50.0)	NC	0	NC
	Man	408 (0.06)	1 (100)	NC	0	NC	0	NC	0	NC
Gardening, landscaping and reforestation companies	Both sexes	44,948 (4.3)	13 (46.4)	0.80 [0.45 - 1.41]	4 (14.3)	1.41 [0.49 - 4.04]	5 (17.9)	0.93 [0.36 - 2.40]	1 (3.6)	NC
	Woman	2,369 (0.7)	1 (14.3)	NC	1 (14.3)	NC	1 (14.3)	NC	1 (14.3)	NC
	Man	42,579 (5.9)	12 (57.1)	0.88 [0.49 - 1.58]	3 (14.3)	1.33 [0.39 - 4.52]	4 (19.0)	1.02 [0.35 - 2.93]	0	NC
Wood production	Both sexes	10,470 (1.0)	6 (60.0)	1.08 [0.48 - 2.43]	0	NC	1 (10.0)	NC	1 (10.0)	NC
	Woman	283 (0.09)	0	NC	0	NC	0	NC	0	NC
	Man	10,187 (1.4)	6 (60.0)	1.25 [0.56 - 2.83]	0	NC	1 (10.0)	NC	1 (10.0)	NC
Shellfish farming	Both sexes	3,350 (0.3)	2 (100)	NC	0	NC	0	NC	0	NC
	Woman	666 (0.2)	0	NC	0	NC	0	NC	0	NC
	Man	2,684 (0.4)	2 (100)	NC	0	NC	0	NC	0	NC
Salt marsh	Both sexes	873 (0.08)	0	NC	0	NC	0	NC	0	NC
	Woman	200 (0.06)	0	NC	0	NC	0	NC	0	NC
	Man	673 (0.09)	0	NC	0	NC	0	NC	0	NC
Fixed sawmill	Both sexes	735 (0.07)	0	NC	0	NC	0	NC	0	NC
	Woman	48 (0.01)	0	NC	0	NC	0	NC	0	NC
	Man	687 (0.1)	0	NC	0	NC	0	NC	0	NC
Rural craftsperson	Both sexes	7,038 (0.7)	0	NC	0	NC	0	NC	0	NC
	Woman	256 (0.08)	0	NC	0	NC	0	NC	0	NC
	Man	6,782 (0.9)	0	NC	0	NC	0	NC	0	NC
Sylviculture/forestry	Both sexes	1,986 (0.2)	2 (100)	NC	0	NC	0	NC	0	NC
	Woman	339 (0.1)	0	NC	0	NC	0	NC	0	NC
	Man	1,647 (0.2)	2 (100)	NC	0	NC	0	NC	0	NC

Note: m: number of exposed cases, NC: not calculated, CNS: central nervous system, C71: malignant neoplasm of brain, C72: malignant neoplasm of spinal cord, cranial nerves and other parts of CNS, D33: benign neoplasm of brain and other parts of CNS, D43: neoplasm of uncertain or unknown behavior of brain and central nervous system, 95% CI: 95% confidence interval.

[a] The percentages in brackets refer to the ratio of exposed cases in the study population and the total number of cases in the overall population.

[b] Hazard ratios were estimated by Cox models with time to first CNS tumor insurance declaration as the underlying timescale, when the number of exposed cases was sufficient ($m \geq 3$), adjusted for sex (for "both sexes" only), age, first year of the farm's establishment, farm surface, earnings, number of associates, unemployment status, total number of farms, family status, partner work status, farm location, number of comorbidities and having a secondary activity.

[c] The percentages in brackets refer to the ratio of exposed cases in the study population and the total number of cases in the study population.

Table S3: Results from sensitivity analysis (age as continuous variable) - Agricultural practices and risks of CNS tumors and meningiomas, TRACTOR, France, 2002-2016

Agricultural practice/activity	Sex	Study population	All CNS tumors		All meningiomas		C70		D32		D42	
		(%)	m[a] (%)	HR[b] [95% CI]	m[c] (%)	HR[b] [95% CI]	m[c] (%)	HR[b] [95% CI]	m[c] (%)	HR[b] [95% CI]	m[c] (%)	HR[b] [95% CI]
Both/mixed dairy and cow farming	Both sexes	30,729 (3.0)	36 (3.5)	0.89 [0.64−1.26]	19 (52.7)	1.56 [0.97−2.50]	5 (13.9)	1.51 [0.60−3.80]	5 (13.9)	0.81 [0.33−2.00]	9 (25.0)	**2.51 [1.25−5.05]**
(individuals performing both dairy and cow farming)	Woman	8,004 (2.5)	15 (3.8)	1.06 [0.63−1.79]	7 (46.7)	1.01 [0.47−2.17]	2 (13.3)	NC	2 (13.3)	NC	3 (20.0)	1.40 [0.43−4.52]
	Man	22,725 (3.2)	21 (3.4)	0.81 [0.52−1.26]	12 (57.1)	**2.22 [1.20−4.09]**	3 (14.3)	1.65 [0.49−5.56]	3 (14.3)	1.16 [0.35−3.79]	6 (28.6)	**4.34 [1.76−10.7]**
Cow farming	Both sexes	110,214 (10.6)	121 (11.9)	0.94 [0.77−1.15]	46 (38.0)	1.16 [0.83−1.60]	9 (7.4)	1.08 [0.53−2.16]	29 (24.0)	**1.60 [1.04−2.45]**	9 (7.4)	0.62 [0.31−1.27]
(individuals performing only cow farming)	Woman	32,699 (10.2)	50 (12.5)	1.06 [0.77−1.44]	30 (60.0)	1.32 [0.87−1.99]	5 (10.0)	1.42 [0.57−3.52]	20 (40.0)	**1.85 [1.11−3.11]**	6 (12.0)	0.73 [0.31−1.75]
	Man	77,515 (10.8)	71 (11.5)	0.86 [0.66−1.12]	16 (22.5)	0.94 [0.54−1.65]	4 (5.6)	0.87 [0.29−2.61]	9 (12.7)	1.42 [0.66−3.05]	3 (4.2)	0.49 [0.14−1.67]
Dairy farming	Both sexes	158,706 (15.3)	186 (18.3)	0.95 [0.80−1.12]	45 (24.2)	**0.69 [0.49−0.95]**	18 (9.7)	1.11 [0.63−1.97]	20 (10.8)	0.62 [0.38−1.02]	12 (6.5)	0.58 [0.31−1.08]
(individuals performing only dairy farming)	Woman	48,823 (15.2)	62 (15.5)	**0.73 [0.55−0.97]**	26 (41.9)	0.66 [0.43−1.03]	10 (16.1)	0.97 [0.45−2.08]	10 (16.1)	**0.49 [0.25−0.97]**	10 (16.1)	0.89 [0.43−1.83]
	Man	109,883 (15.4)	124 (20.0)	1.12 [0.90−1.38]	19 (15.3)	0.67 [0.40−1.11]	8 (6.5)	1.15 [0.48−2.77]	10 (8.1)	0.80 [0.38−1.66]	2 (1.6)	NC
Ovine and caprine farming	Both sexes	47,086 (4.5)	45 (4.4)	0.98 [0.72−1.33]	16 (35.6)	1.08 [0.65−1.80]	2 (4.4)	NC	6 (13.3)	0.84 [0.37−1.93]	8 (17.8)	1.66 [0.79−3.46]
	Woman	16,808 (5.2)	16 (4.0)	0.75 [0.45−1.25]	9 (56.3)	0.87 [0.44−1.71]	1 (6.3)	NC	4 (25.0)	0.72 [0.26−1.99]	4 (25.0)	1.30 [0.46−3.65]
	Man	30,278 (4.2)	29 (4.7)	1.20 [0.82−1.76]	7 (24.1)	1.58 [0.72−3.44]	1 (3.4)	NC	2 (6.7)	NC	4 (4.7)	2.26 [0.80−6.36]
Pig farming	Both sexes	13,389 (1.3)	24 (2.4)	**1.55 [1.03−2.32]**	6 (25.0)	1.41 [0.61−3.24]	1 (4.2)	NC	1 (4.2)	NC	4 (16.7)	2.72 [0.95−7.76]
	Woman	3,830 (1.2)	5 (1.3)	0.82 [0.33−2.03]	2 (40.0)	NC	0	NC	1 (20.0)	NC	1 (20.0)	NC
	Man	9,559 (1.3)	19 (3.1)	**1.95 [1.21−3.14]**	4 (21.0)	2.27 [0.79−6.44]	1 (5.3)	NC	0	NC	3 (15.8)	**6.03 [1.75−20.8]**
Poultry and rabbit farming	Both sexes	24,576 (2.4)	25 (2.5)	0.92 [0.62−1.38]	6 (24.0)	0.66 [0.29−1.49]	1 (4.0)	NC	2 (8.0)	NC	3 (12.0)	0.96 [0.30−3.08]
	Woman	9,671 (3.0)	14 (3.5)	1.01 [0.58−1.72]	4 (28.6)	0.60 [0.22−1.64]	1 (7.1)	NC	1 (7.1)	NC	2 (14.3)	NC
	Man	14,905 (2.1)	11 (1.8)	0.79 [0.43−1.45]	2 (18.2)	NC	0	NC	1 (9.1)	NC	1 (9.1)	NC
Stud farming	Both sexes	15,641 (1.5)	6 (0.6)	0.65 [0.29−1.46]	3 (50.0)	0.95 [0.30−3.00]	0	NC	3 (50.0)	1.89 [0.59−6.01]	0	NC
	Woman	6,831 (2.1)	5 (1.3)	0.87 [0.36−2.15]	2 (40.0)	NC	0	NC	2 (40.0)	NC	0	NC
	Man	8,810 (1.2)	1 (0.2)	NC	1 (100)	NC	0	NC	1 (100)	NC	0	NC
Training, dressage, riding clubs	Both sexes	13,273 (1.3)	8 (0.8)	0.67 [0.33−1.36]	2 (25.0)	NC	1 (12.5)	NC	0	NC	1 (12.5)	NC
	Woman	6,049 (1.9)	2 (0.5)	NC	1 (50.0)	NC	1 (50.0)	NC	0	NC	0	NC
	Man	7,224 (1.0)	6 (1.0)	1.10 [0.49−2.47]	1 (16.7)	NC	0	NC	0	NC	1 (16.7)	NC
Unspecified large animal farming	Both sexes	2,663 (0.3)	4 (0.4)	2.30 [0.86−6.15]	2 (50.0)	NC	1 (25.0)	NC	1 (25.0)	NC	0	NC
(e.g. large dogs, zoo)	Woman	1,280 (0.4)	3 (0.8)	2.91 [0.93−9.08]	2 (66.7)	NC	1 (33.3)	NC	1 (33.3)	NC	0	NC
	Man	1,383 (0.2)	1 (0.2)	NC	0	NC	0	NC	0	NC	0	NC
Unspecified small animal farming	Both sexes	18,058 (1.7)	10 (1.0)	1.16 [0.62−2.16]	3 (30.0)	1.11 [0.34−3.60]	0	NC	2 (20.0)	NC	1 (10.0)	NC
(e.g. frogs, snails, bees)	Woman	7,698 (2.4)	5 (1.3)	1.21 [0.49−2.93]	1 (20.0)	NC	0	NC	2 (40.0)	NC	0	NC
	Man	10,360 (1.4)	5 (0.8)	1.11 [0.45−2.67]	1 (20.0)	NC	0	NC	0	NC	1 (20.0)	NC
Fruit arboriculture	Both sexes	24,086 (2.3)	27 (2.7)	**1.50 [1.02−2.22]**	3 (11.1)	0.53 [0.17−1.67]	0	NC	2 (7.4)	NC	1 (3.7)	NC
	Woman	7,649 (2.4)	10 (2.5)	1.30 [0.68−2.47]	2 (20.0)	NC	0	NC	1 (10.0)	NC	1 (10.0)	NC
	Man	16,437 (2.3)	17 (1.1)	1.49 [0.91−2.42]	1 (5.9)	NC	0	NC	1 (5.9)	NC	0	NC
Garden center	Both sexes	5,111 (0.5)	3 (0.3)	0.77 [0.24−2.40]	2 (66.7)	NC	0	NC	2 (66.7)	NC	0	NC
	Woman	1,358 (0.4)	2 (0.5)	NC	2 (100)	NC	0	NC	2 (100)	NC	0	NC
	Man	3,753 (0.5)	1 (0.2)	NC	0	NC	0	NC	0	NC	0	NC
Truck farming, floriculture/flower-growing	Both sexes	41,525 (4.0)	44 (4.3)	**1.45 [1.06−1.98]**	14 (31.8)	1.69 [0.97−2.94]	3 (6.8)	1.86 [0.56−6.13]	8 (18.2)	1.98 [0.94−4.16]	3 (6.8)	0.96 [0.29−3.11]
	Woman	12,672 (4.0)	17 (4.3)	1.41 [0.85−2.34]	8 (47.0)	1.50 [0.72−3.11]	2 (11.8)	NC	4 (23.5)	1.39 [0.49−3.89]	2 (11.8)	NC
	Man	28,853 (4.0)	27 (4.4)	1.46 [0.98−2.19]	6 (22.2)	1.96 [0.83−4.60]	1 (3.7)	NC	4 (14.8)	**3.08 [1.04−9.11]**	1 (3.7)	NC
Unspecified and mixed farming	Both sexes	120,746 (11.7)	140 (13.8)	**1.24 [1.01−1.53]**	36 (25.7)	1.01 [0.68−1.49]	7 (5.0)	0.69 [0.28−1.70]	12 (8.6)	0.59 [0.30−1.13]	20 (14.3)	**2.03 [1.16−3.54]**
(e.g. polyculture, mixed farming)	Woman	36,955 (11.5)	59 (14.8)	**1.48 [1.08−2.03]**	25 (42.4)	1.26 [0.78−2.02]	4 (6.8)	0.70 [0.22−2.25]	9 (15.3)	0.69 [0.32−1.47]	14 (23.7)	**2.74 [1.39−5.41]**
	Man	83,791 (11.7)	81 (13.1)	1.10 [0.84−1.45]	11 (13.6)	0.67 [0.33−1.35]	3 (3.7)	0.67 [0.16−2.77]	3 (3.7)	0.33 [0.09−1.19]	6 (7.4)	1.23 [0.46−3.29]
Unspecified specialized crop farming	Both sexes	6,168 (0.6)	4 (0.4)	1.04 [0.38−2.78]	2 (50.0)	NC	0	NC	2 (50.0)	NC	0	NC
(e.g. horticulture)	Woman	2,231 (0.7)	2 (0.5)	NC	1 (50.0)	NC	0	NC	1 (50.0)	NC	0	NC
	Man	3,933 (0.5)	2 (0.3)	NC	1 (50.0)	NC	0	NC	1 (50.0)	NC	0	NC
Viticulture	Both sexes	118,577 (11.4)	113 (11.1)	1.12 [0.88−1.40]	40 (35.4)	1.41 [0.95−2.09]	6 (5.3)	0.73 [0.28−1.89]	22 (19.5)	1.63 [0.95−2.79]	13 (11.5)	1.25 [0.63−2.49]
	Woman	41,970 (13.1)	49 (12.3)	1.10 [0.77−1.57]	27 (55.1)	1.36 [0.83−2.21]	6 (12.2)	1.93 [0.67−5.54]	15 (30.6)	1.56 [0.81−2.98]	7 (14.3)	0.98 [0.39−2.49]
	Man	76,607 (10.7)	64 (10.4)	1.12 [0.82−1.51]	13 (20.3)	1.49 [0.75−2.94]	0	NC	7 (10.9)	2.16 [0.82−5.66]	6 (9.4)	1.71 [0.61−4.81]
Crop farming (including field crops, cereal grain crops,	Both sexes	305,838 (29.5)	258 (25.4)	**1.58 [1.35−1.86]**	87 (33.7)	**1.67 [1.25−2.22]**	19 (7.4)	1.55 [0.84−2.83]	46 (17.8)	**1.98 [1.32−2.96]**	29 (11.2)	1.45 [0.88−2.37]

wheat and industrial grower)	Woman	102,240 (31.9)	113 (28.3)	**1.83 [1.43−2.36]**	55 (48.7)	**1.76 [1.22−2.53]**	11 (9.7)	1.85 [0.82−4.15]	32 (28.3)	**1.98 [1.21−3.23]**	17 (15.0)	1.33 [0.70−2.52]
	Man	203,598 (28.4)	145 (23.5)	**1.40 [1.13−1.74]**	32 (22.1)	1.59 [0.99−2.54]	8 (5.5)	1.28 [0.50−3.27]	14 (9.7)	1.88 [0.91−3.87]	12 (8.3)	1.72 [0.78−3.76]
Agricultural work companies	Both sexes	14,282 (1.4)	11 (1.1)	1.21 [0.65−2.21]	4 (36.4)	1.83 [0.66−5.08]	0	NC	1 (9.1)	NC	3 (27.3)	**3.74 [1.17−12.0]**
	Woman	1,715 (0.5)	0	NC	0	NC	0	NC	0	NC	0	NC
	Man	12,567 (1.8)	11 (1.8)	1.33 [0.73−2.43]	4 (36.4)	2.59 [0.94−7.07]	0	NC	1 (9.1)	NC	3 (27.3)	**5.05 [1.54−16.6]**
Company representative/authorized representative	Both sexes	1,846 (0.2)	3 (0.3)	3.09 [0.99−9.63]	1 (33.3)	NC	0	NC	1 (33.3)	NC	0	NC
	Woman	1,435 (0.4)	2 (0.5)	NC	1 (50.0)	NC	0	NC	1 (50.0)	NC	0	NC
	Man	408 (0.06)	1 (0.2)	NC	0	NC	0	NC	0	NC	0	NC
Gardening, landscaping and reforestation companies	Both sexes	44,948 (4.3)	28 (2.8)	1.07 [0.67−1.70]	5 (17.9)	0.77 [0.28−2.11]	3 (10.7)	2.86 [0.87−9.42]	2 (7.1)	NC	0	NC
	Woman	2,369 (0.7)	7 (1.8)	**3.42 [1.61−7.27]**	3 (42.9)	**3.35 [1.06−10.6]**	2 (28.6)	NC	1 (14.3)	NC	0	NC
	Man	42,579 (5.9)	21 (3.4)	0.75 [0.48−1.17]	2 (9.5)	NC	1 (4.8)	NC	1 (4.8)	NC	0	NC
Wood production	Both sexes	10,470 (1.0)	10 (1.0)	1.13 [0.60−2.12]	2 (20.0)	NC	0	NC	1 (10.0)	NC	1 (10.0)	NC
	Woman	283 (0.09)	0	NC	0	NC	0	NC	0	NC	0	NC
	Man	10,187 (1.4)	10 (1.6)	1.17 [0.62−2.20]	2 (20.0)	NC	0	NC	1 (10.0)	NC	1 (10.0)	NC
Shellfish farming	Both sexes	3,350 (0.3)	2 (0.2)	NC	0	NC	0	NC	0	NC	0	NC
	Woman	666 (0.2)	0	NC	0	NC	0	NC	0	NC	0	NC
	Man	2,684 (0.4)	2 (0.3)	NC	0	NC	0	NC	0	NC	0	NC
Salt marsh	Both sexes	873 (0.08)	0	NC	0	NC	0	NC	0	NC	0	NC
	Woman	200 (0.06)	0	NC	0	NC	0	NC	0	NC	0	NC
	Man	673 (0.09)	0	NC	0	NC	0	NC	0	NC	0	NC
Fixed sawmill	Both sexes	735 (0.07)	0	NC	0	NC	0	NC	0	NC	0	NC
	Woman	48 (0.01)	0	NC	0	NC	0	NC	0	NC	0	NC
	Man	687 (0.1)	0	NC	0	NC	0	NC	0	NC	0	NC
Rural craftsperson	Both sexes	7,038 (0.7)	0	NC	0	NC	0	NC	0	NC	0	NC
	Woman	256 (0.08)	0	NC	0	NC	0	NC	0	NC	0	NC
	Man	6,782 (0.9)	0	NC	0	NC	0	NC	0	NC	0	NC
Sylviculture/forestry	Both sexes	1,986 (0.2)	2 (0.2)	NC	0	NC	0	NC	0	NC	0	NC
	Woman	339 (0.1)	0	NC	0	NC	0	NC	0	NC	0	NC
	Man	1,647 (0.2)	2 (0.3)	NC	0	NC	0	NC	0	NC	0	NC

Note: m: number of exposed cases, NC: not calculated, CNS: central nervous system, C70: malignant neoplasm of meninges, D32: benign neoplasm of meninges, D42: neoplasm of uncertain or unknown behavior of meninges, 95% CI: 95% confidence interval.

[a] The percentages in brackets refer to the ratio of exposed cases in the study population and the total number of cases in the overall population.

[b] Hazard ratios were estimated by Cox models with time to first CNS tumor insurance declaration as the underlying timescale, when the number of exposed cases was sufficient ($m \geq 3$), adjusted for sex (for “both sexes” only), age, first year of the farm’s establishment, farm surface, earnings, number of associates, unemployment status, total number of farms, family status, partner work status, farm location, number of comorbidities and having a secondary activity.

[c] The percentages in brackets refer to the ratio of exposed cases in the study population and the total number of cases in the study population.

Table S4: Results from sensitivity analysis (age as continuous variable) - Agricultural practices and risks of gliomas and tumors of spinal cord, cranial nerves and other parts of CNS, TRACTOR, France, 2002-2016

Agricultural practice/activity	Sex	Study population (%)	C71		D33		D43		C72	
			m[c] (%)	HR[b] [95% CI]	m[c] (%)	HR[b] [95% CI]	m[c] (%)	HR[b] [95% CI]	m[c] (%)	HR[b] [95% CI]
Both/mixed dairy and cow farming (individuals performing both dairy and cow farming)	Both sexes	30,729 (3.0)	11 (30.6)	0.55 [0.30−1.01]	1 (2.8)	NC	5 (13.9)	1.32 [0.52−3.27]	0	NC
	Woman	8,004 (2.5)	5 (33.3)	1.15 [0.46−2.85]	0	NC	3 (20.0)	2.64 [0.79−8.79]	0	NC
	Man	22,725 (3.2)	6 (28.6)	**0.39 [0.17−0.87]**	1 (4.8)	NC	2 (9.5)	NC	0	NC
Cow farming (individuals performing only cow farming)	Both sexes	110,214 (10.6)	45 (37.2)	**0.69 [0.50−0.95]**	7 (5.8)	0.61 [0.27−1.35]	21 (17.4)	**2.02 [1.21−3.39]**	2 (1.7)	NC
	Woman	32,699 (10.2)	10 (20.0)	0.64 [0.33−1.26]	3 (6.0)	0.65 [0.19−2.19]	7 (14.0)	1.91 [0.79−4.56]	0	NC
	Man	77,515 (10.8)	35 (49.3)	**0.68 [0.47−0.98]**	4 (5.6)	0.59 [0.20−1.70]	14 (19.7)	**2.11 [1.11−4.02]**	2 (2.8)	NC
Dairy farming (individuals performing only dairy farming)	Both sexes	158,706 (15.3)	99 (53.2)	1.08 [0.85−1.37]	16 (8.6)	1.16 [0.65−2.05]	16 (8.6)	0.73 [0.42−1.29]	13 (7.0)	**2.08 [1.03−4.19]**
	Woman	48,823 (15.2)	18 (29.0)	0.60 [0.35−1.03]	8 (12.9)	1.32 [0.56−3.11]	6 (9.7)	0.70 [0.27−1.79]	6 (9.7)	2.53 [0.85−7.48]
	Man	109,883 (15.4)	81 (65.3)	**1.32 [1.02−1.71]**	8 (6.5)	1.24 [0.55−2.75]	10 (8.1)	0.77 [0.38−1.58]	7 (5.6)	1.77 [0.67−4.60]
Ovine and caprine farming	Both sexes	47,086 (4.5)	23 (51.1)	1.08 [0.70−1.64]	4 (8.9)	1.07 [0.38−2.94]	4 (8.9)	0.75 [0.27−2.06]	0	NC
	Woman	16,808 (5.2)	4 (25.0)	0.58 [0.21−1.61]	3 (18.8)	1.79 [0.53−5.97]	1 (6.3)	NC	0	NC
	Man	30,278 (4.2)	19 (65.5)	1.33 [0.83−2.13]	1 (3.4)	NC	3 (10.3)	0.91 [0.28−2.97]	0	NC
Pig farming	Both sexes	13,389 (1.3)	17 (70.8)	**2.06 [1.24−3.41]**	0	NC	0	NC	1 (4.2)	NC
	Woman	3,830 (1.2)	3 (60.0)	1.44 [0.44−4.70]	0	NC	0	NC	0	NC
	Man	9,559 (1.3)	14 (73.7)	**2.27 [1.29−3.99]**	0	NC	0	NC	1 (5.3)	NC
Poultry and rabbit farming	Both sexes	24,576 (2.4)	13 (52.0)	0.99 [0.57−1.73]	3 (12.0)	1.43 [0.44−4.59]	2 (8.0)	NC	2 (8.0)	NC
	Woman	9,671 (3.0)	8 (57.1)	1.85 [0.89−3.85]	1 (7.1)	NC	0	NC	2 (14.3)	NC
	Man	14,905 (2.1)	5 (45.5)	0.58 [0.24−1.42]	2 (18.2)	NC	2 (18.2)	NC	0	NC
Stud farming	Both sexes	15,641 (1.5)	3 (50.0)	0.69 [0.22−2.19]	0	NC	0	NC	0	NC
	Woman	6,831 (2.1)	3 (60.0)	1.44 [0.44−4.67]	0	NC	0	NC	0	NC
	Man	8,810 (1.2)	0	NC	0	NC	0	NC	0	NC
Training, dressage, riding clubs	Both sexes	13,273 (1.3)	4 (50.0)	0.76 [0.28−2.04]	0	NC	2 (25.0)	NC	0	NC
	Woman	6,049 (1.9)	1 (50.0)	NC	0	NC	0	NC	0	NC
	Man	7,224 (1.0)	3 (50.0)	0.96 [0.30−3.01]	0	NC	2 (33.3)	NC	0	NC
Unspecified large animal farming (e.g. large dogs, zoo)	Both sexes	2,663 (0.3)	1 (25.0)	NC	0	NC	1 (25.0)	NC	0	NC
	Woman	1,280 (0.4)	0	NC	0	NC	1 (33.3)	NC	0	NC
	Man	1,383 (0.2)	1 (100)	NC	0	NC	0	NC	0	NC
Unspecified small animal farming (e.g. frogs, snails, bees)	Both sexes	18,058 (1.7)	4 (40.0)	1.04 [0.38−2.77]	1 (10.0)	NC	2 (20.0)	NC	0	NC
	Woman	7,698 (2.4)	1 (20.0)	NC	1 (20.0)	NC	1 (20.0)	NC	0	NC
	Man	10,360 (1.4)	3 (60.0)	1.15 [0.36−3.59]	0	NC	1 (20.0)	NC	0	NC
Fruit arboriculture	Both sexes	24,086 (2.3)	14 (51.9)	1.61 [0.93−2.77]	3 (11.1)	1.85 [0.56−5.98]	6 (22.2)	**3.38 [1.46−7.83]**	2 (7.4)	NC
	Woman	7,649 (2.4)	6 (60.0)	2.33 [0.99−5.44]	1 (10.0)	NC	1 (10.0)	NC	0	NC
	Man	16,437 (2.3)	8 (47.1)	1.25 [0.61−2.55]	2 (11.8)	NC	5 (29.4)	**4.08 [1.57−10.6]**	2 (11.8)	NC
Garden center	Both sexes	5,111 (0.5)	0	NC	1 (33.3)	NC	0	NC	0	NC
	Woman	1,358 (0.4)	0	NC	0	NC	0	NC	0	NC
	Man	3,753 (0.5)	0	NC	1 (100)	NC	0	NC	0	NC
Truck farming, floriculture/flower-growing	Both sexes	41,525 (4.0)	17 (38.6)	1.06 [0.64−1.76]	3 (6.8)	1.05 [0.32−3.45]	6 (13.6)	1.90 [0.80−4.48]	4 (9.1)	**3.91 [1.31−11.6]**
	Woman	12,672 (4.0)	5 (29.4)	1.15 [0.45−2.90]	1 (5.9)	NC	2 (11.8)	NC	1 (5.9)	NC
	Man	28,853 (4.0)	12 (44.4)	1.03 [0.57−1.88]	2 (7.4)	NC	4 (14.8)	1.81 [0.62−5.19]	3 (11.1)	**6.75 [1.81−25.2]**
Unspecified and mixed farming (e.g. polyculture, mixed farming)	Both sexes	120,746 (11.7)	72 (51.4)	1.29 [0.96−1.74]	13 (9.3)	1.16 [0.57−2.33]	17 (12.1)	2.00 [1.12−3.59]	6 (4.3)	1.10 [0.39−3.02]
	Woman	36,955 (11.5)	19 (32.2)	1.29 [0.72−2.31]	5 (8.5)	1.62 [0.54−4.78]	7 (11.9)	2.70 [1.09−6.70]	5 (8.5)	3.14 [0.91−10.8]
	Man	83,791 (11.7)	53 (65.4)	1.29 [0.91−1.83]	8 (9.9)	0.99 [0.39−2.50]	10 (12.3)	1.64 [0.76−3.52]	1 (1.2)	NC
Unspecified specialized crop farming (e.g. horticulture)	Both sexes	6,168 (0.6)	1 (25.0)	NC	1 (25.0)	NC	0	NC	0	NC
	Woman	2,231 (0.7)	1 (50.0)	NC	0	NC	0	NC	0	NC
	Man	3,933 (0.5)	0	NC	1 (50.0)	NC	0	NC	0	NC
Viticulture	Both sexes	118,577 (11.4)	53 (46.9)	1.09 [0.78−1.53]	12 (10.6)	1.20 [0.58−2.45]	8 (7.1)	0.76 [0.34−1.70]	1 (0.9)	NC
	Woman	41,970 (13.1)	17 (34.7)	1.11 [0.59−2.05]	3 (6.1)	0.84 [0.22−3.18]	2 (4.1)	NC	0	NC
	Man	76,607 (10.7)	36 (56.3)	1.08 [0.72−1.62]	9 (14.1)	1.50 [0.63−3.54]	6 (9.4)	0.91 [0.35−2.37]	1 (1.6)	NC
Crop farming (including field crops, cereal grain crops,	Both sexes	305,838 (29.5)	116 (45.0)	**1.52 [1.20−1.93]**	29 (11.2)	**2.54 [1.50−4.33]**	21 (8.1)	1.10 [0.63−1.88]	11 (4.3)	1.53 [0.69−3.35]

wheat and industrial grower)	Woman	102,240 (31.9)	36 (31.9)	**2.02 [1.29−3.17]**	11 (9.7)	**2.53 [1.13−5.69]**	10 (8.8)	1.67 [0.73−3.79]	4 (3.5)	0.93 [0.26−3.25]
	Man	203,598 (28.4)	80 (55.2)	1.33 [1.00−1.78]	18 (12.4)	**2.29 [1.11−4.70]**	11 (7.6)	0.79 [0.38−1.65]	7 (4.8)	2.18 [0.78−6.03]
Agricultural work companies	Both sexes	14,282 (1.4)	4 (36.4)	0.77 [0.28−2.08]	2 (18.2)	NC	1 (9.1)	NC	0	NC
	Woman	1,715 (0.5)	0	NC	0	NC	0	NC	0	NC
	Man	12,567 (1.8)	4 (36.4)	0.84 [0.31−2.26]	2 (18.2)	NC	1 (9.1)	NC	0	NC
Company representative/authorized representative	Both sexes	1,846 (0.2)	1 (33.3)	NC	0	NC	1 (33.3)	NC	0	NC
	Woman	1,435 (0.4)	0	NC	0	NC	1 (50.0)	NC	0	NC
	Man	408 (0.06)	1 (100)	NC	0	NC	0	NC	0	NC
Gardening, landscaping and reforestation companies	Both sexes	44,948 (4.3)	13 (46.4)	1.06 [0.53−2.11]	4 (14.3)	1.36 [0.48−3.82]	5 (17.9)	1.18 [0.37−3.76]	1 (3.6)	NC
	Woman	2,369 (0.7)	1 (14.3)	NC	1 (14.3)	NC	1 (14.3)	NC	1 (14.3)	NC
	Man	42,579 (5.9)	12 (57.1)	0.75 [0.42−1.36]	3 (14.3)	1.53 [0.46−5.01]	4 (19.0)	1.21 [0.32−4.53]	0	NC
Wood production	Both sexes	10,470 (1.0)	6 (60.0)	1.68 [0.70−4.01]	0	NC	1 (10.0)	NC	1 (10.0)	NC
	Woman	283 (0.09)	0	NC	0	NC	0	NC	0	NC
	Man	10,187 (1.4)	6 (60.0)	1.31 [0.58−2.95]	0	NC	1 (10.0)	NC	1 (10.0)	NC
Shellfish farming	Both sexes	3,350 (0.3)	2 (100)	NC	0	NC	0	NC	0	NC
	Woman	666 (0.2)	0	NC	0	NC	0	NC	0	NC
	Man	2,684 (0.4)	2 (100)	NC	0	NC	0	NC	0	NC
Salt marsh	Both sexes	873 (0.08)	0	NC	0	NC	0	NC	0	NC
	Woman	200 (0.06)	0	NC	0	NC	0	NC	0	NC
	Man	673 (0.09)	0	NC	0	NC	0	NC	0	NC
Fixed sawmill	Both sexes	735 (0.07)	0	NC	0	NC	0	NC	0	NC
	Woman	48 (0.01)	0	NC	0	NC	0	NC	0	NC
	Man	687 (0.1)	0	NC	0	NC	0	NC	0	NC
Rural craftsperson	Both sexes	7,038 (0.7)	0	NC	0	NC	0	NC	0	NC
	Woman	256 (0.08)	0	NC	0	NC	0	NC	0	NC
	Man	6,782 (0.9)	0	NC	0	NC	0	NC	0	NC
Sylviculture/forestry	Both sexes	1,986 (0.2)	2 (100)	NC	0	NC	0	NC	0	NC
	Woman	339 (0.1)	0	NC	0	NC	0	NC	0	NC
	Man	1,647 (0.2)	2 (100)	NC	0	NC	0	NC	0	NC

Note: m: number of exposed cases, NC: not calculated, CNS: central nervous system, C71: malignant neoplasm of brain, C72: malignant neoplasm of spinal cord, cranial nerves and other parts of CNS, D33: benign neoplasm of brain and other parts of CNS, D43: neoplasm of uncertain or unknown behavior of brain and central nervous system, 95% CI: 95% confidence interval.

[a] The percentages in brackets refer to the ratio of exposed cases in the study population and the total number of cases in the overall population.

[b] Hazard ratios were estimated by Cox models with time to first CNS tumor insurance declaration as the underlying timescale, when the number of exposed cases was sufficient ($m \geq 3$), adjusted for sex (for "both sexes" only), age, first year of the farm's establishment, farm surface, earnings, number of associates, unemployment status, total number of farms, family status, partner work status, farm location, number of comorbidities and having a secondary activity.

[c] The percentages in brackets refer to the ratio of exposed cases in the study population and the total number of cases in the study population.

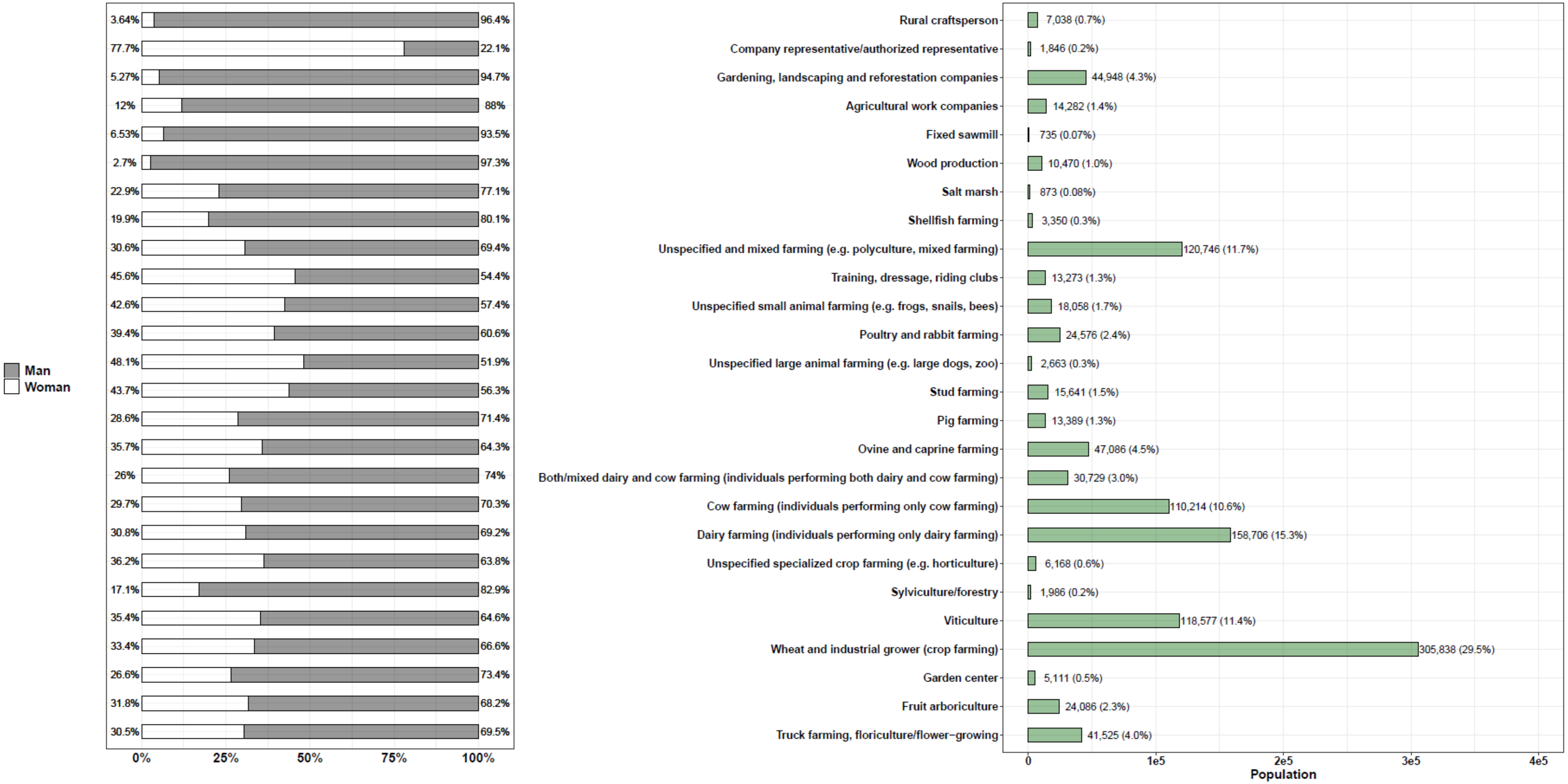

Fig. S1: Number of farm managers by agricultural activities and sex

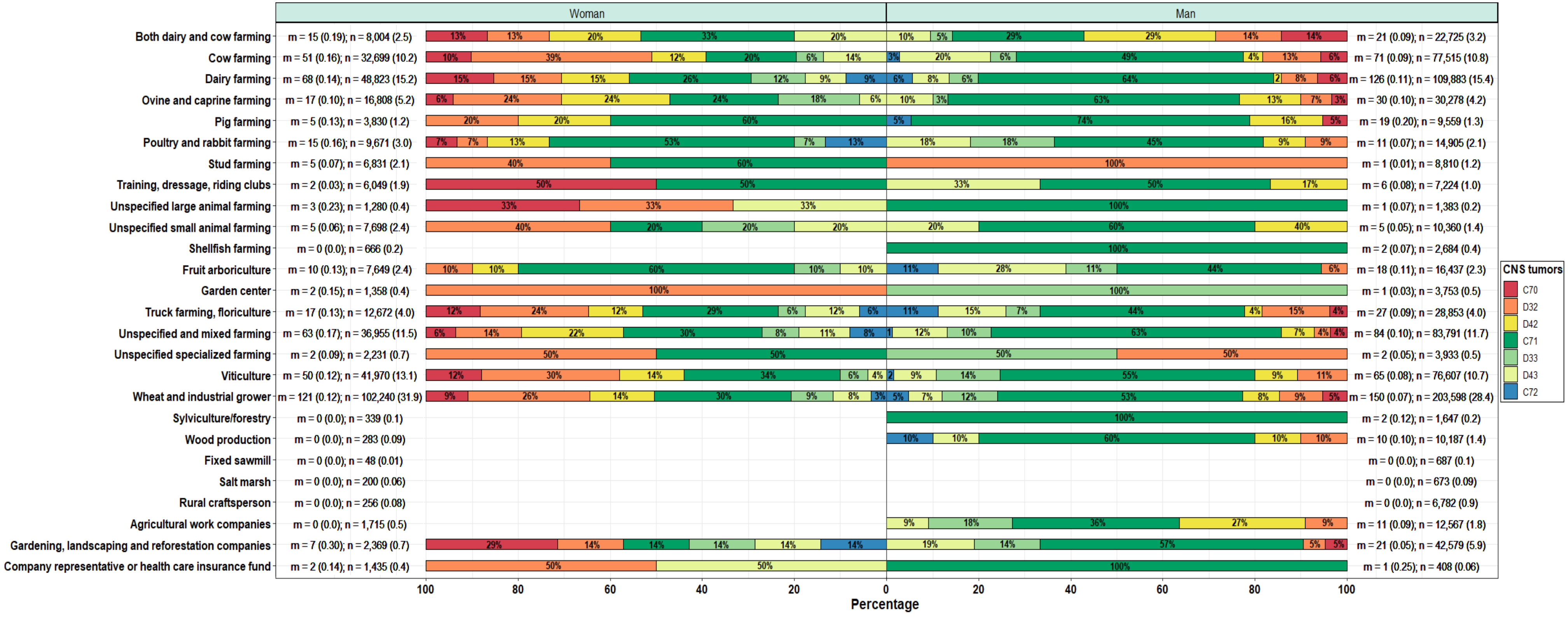

Fig. S2: Percentage of CNS tumors by sex, activity and CNS type

CNS: central nervous system, C70: malignant neoplasm of meninges, C71: malignant neoplasm of brain, C72: malignant neoplasm of spinal cord, cranial nerves and other parts of CNS, D32: benign neoplasm of meninges, D33: benign neoplasm of brain and other parts of CNS, D42: neoplasm of uncertain or unknown behavior of meninges, D43: neoplasm of uncertain or unknown behavior of brain and central nervous system.

	All CNS tumors	All meningiomas	C70	D32	D42	C71	D33	D43	C72
Rural craftsperson	Not calculated<br>n=7038, m=0	Not calculated<br>n=7038, m=0	Not calculated<br>n=7038, m=0	Not calculated<br>n=7038, m=0	Not calculated<br>n=7038, m=0	Not calculated<br>n=7038, m=0	Not calculated<br>n=7038, m=0	Not calculated<br>n=7038, m=0	Not calculated<br>n=7038, m=0
Company representative/authorized representative	4.15 [1.33 – 12.9]<br>n=1846, m=3	Not calculated<br>n=1846, m=1	Not calculated<br>n=1846, m=0	Not calculated<br>n=1846, m=1	Not calculated<br>n=1846, m=0	Not calculated<br>n=1846, m=1	Not calculated<br>n=1846, m=0	Not calculated<br>n=1846, m=1	Not calculated<br>n=1846, m=0
Gardening, landscaping and reforestation companies	0.87 [0.59 – 1.28]<br>n=44948, m=28	0.59 [0.24 – 1.45]<br>n=44948, m=5	2.23 [0.68 – 7.26]<br>n=44948, m=3	Not calculated<br>n=44948, m=2	Not calculated<br>n=44948, m=0	0.80 [0.45 – 1.41]<br>n=44948, m=13	1.41 [0.49 – 4.04]<br>n=44948, m=4	0.93 [0.36 – 2.40]<br>n=44948, m=5	Not calculated<br>n=44948, m=1
Agricultural work companies	1.04 [0.58 – 1.90]<br>n=14282, m=11	1.35 [0.50 – 3.62]<br>n=14282, m=4	Not calculated<br>n=14282, m=0	Not calculated<br>n=14282, m=1	3.05 [0.96 – 9.68]<br>n=14282, m=3	0.77 [0.29 – 2.07]<br>n=14282, m=4	Not calculated<br>n=14282, m=2	Not calculated<br>n=14282, m=1	Not calculated<br>n=14282, m=0
Fixed sawmill	Not calculated<br>n=735, m=0	Not calculated<br>n=735, m=0	Not calculated<br>n=735, m=0	Not calculated<br>n=735, m=0	Not calculated<br>n=735, m=0	Not calculated<br>n=735, m=0	Not calculated<br>n=735, m=0	Not calculated<br>n=735, m=0	Not calculated<br>n=735, m=0
Wood production	0.83 [0.44 – 1.55]<br>n=10470, m=10	Not calculated<br>n=10470, m=2	Not calculated<br>n=10470, m=0	Not calculated<br>n=10470, m=1	Not calculated<br>n=10470, m=1	1.08 [0.48 – 2.43]<br>n=10470, m=6	Not calculated<br>n=10470, m=0	Not calculated<br>n=10470, m=1	Not calculated<br>n=10470, m=1
Salt marsh	Not calculated<br>n=873, m=0	Not calculated<br>n=873, m=0	Not calculated<br>n=873, m=0	Not calculated<br>n=873, m=0	Not calculated<br>n=873, m=0	Not calculated<br>n=873, m=0	Not calculated<br>n=873, m=0	Not calculated<br>n=873, m=0	Not calculated<br>n=873, m=0
Shellfish farming	Not calculated<br>n=3350, m=2	Not calculated<br>n=3350, m=0	Not calculated<br>n=3350, m=0	Not calculated<br>n=3350, m=0	Not calculated<br>n=3350, m=0	Not calculated<br>n=3350, m=2	Not calculated<br>n=3350, m=0	Not calculated<br>n=3350, m=0	Not calculated<br>n=3350, m=0
Unspecified and mixed farming (e.g. polyculture, mixed farming)	1.34 [1.12 – 1.61]<br>n=120746, m=140	1.10 [0.77 – 1.56]<br>n=120746, m=36	0.88 [0.40 – 1.94]<br>n=120746, m=7	0.72 [0.40 – 1.31]<br>n=120746, m=12	1.83 [1.11 – 3.03]<br>n=120746, m=20	1.52 [1.17 – 1.96]<br>n=120746, m=72	1.47 [0.80 – 2.70]<br>n=120746, m=13	1.48 [0.87 – 2.52]<br>n=120746, m=17	1.04 [0.43 – 2.53]<br>n=120746, m=6
Training, dressage, riding clubs	0.91 [0.45 – 1.83]<br>n=13273, m=8	Not calculated<br>n=13273, m=2	Not calculated<br>n=13273, m=1	Not calculated<br>n=13273, m=0	Not calculated<br>n=13273, m=1	0.83 [0.31 – 2.24]<br>n=13273, m=4	Not calculated<br>n=13273, m=0	Not calculated<br>n=13273, m=2	Not calculated<br>n=13273, m=0
Unspecified small animal farming (e.g. frogs, snails, bees)	1.56 [0.84 – 2.91]<br>n=18058, m=10	1.54 [0.49 – 4.80]<br>n=18058, m=3	Not calculated<br>n=18058, m=0	Not calculated<br>n=18058, m=2	Not calculated<br>n=18058, m=1	1.31 [0.49 – 3.52]<br>n=18058, m=4	Not calculated<br>n=18058, m=1	Not calculated<br>n=18058, m=2	Not calculated<br>n=18058, m=0
Poultry and rabbit farming	1.13 [0.76 – 1.69]<br>n=24576, m=25	0.93 [0.41 – 2.09]<br>n=24576, m=6	Not calculated<br>n=24576, m=1	Not calculated<br>n=24576, m=2	1.36 [0.42 – 4.34]<br>n=24576, m=3	1.14 [0.65 – 2.00]<br>n=24576, m=13	1.72 [0.53 – 5.55]<br>n=24576, m=3	Not calculated<br>n=24576, m=2	Not calculated<br>n=24576, m=2
Unspecified large animal farming (e.g. large dogs, zoo)	3.67 [1.37 – 9.82]<br>n=2663, m=4	Not calculated<br>n=2663, m=2	Not calculated<br>n=2663, m=1	Not calculated<br>n=2663, m=1	Not calculated<br>n=2663, m=0	Not calculated<br>n=2663, m=1	Not calculated<br>n=2663, m=0	Not calculated<br>n=2663, m=1	Not calculated<br>n=2663, m=0
Stud farming	0.85 [0.38 – 1.91]<br>n=15641, m=6	1.53 [0.49 – 4.81]<br>n=15641, m=3	Not calculated<br>n=15641, m=0	3.16 [0.99 – 10.0]<br>n=15641, m=3	Not calculated<br>n=15641, m=0	0.84 [0.27 – 2.62]<br>n=15641, m=3	Not calculated<br>n=15641, m=0	Not calculated<br>n=15641, m=0	Not calculated<br>n=15641, m=0
Pig farming	1.67 [1.10 – 2.54]<br>n=13389, m=24	1.58 [0.68 – 3.64]<br>n=13389, m=6	Not calculated<br>n=13389, m=1	Not calculated<br>n=13389, m=1	3.18 [1.11 – 9.16]<br>n=13389, m=4	2.28 [1.37 – 3.80]<br>n=13389, m=17	Not calculated<br>n=13389, m=0	Not calculated<br>n=13389, m=0	Not calculated<br>n=13389, m=1
Ovine and caprine farming	1.17 [0.86 – 1.58]<br>n=47086, m=45	1.47 [0.88 – 2.45]<br>n=47086, m=16	Not calculated<br>n=47086, m=2	1.10 [0.48 – 2.52]<br>n=47086, m=6	2.27 [1.08 – 4.76]<br>n=47086, m=8	1.26 [0.82 – 1.93]<br>n=47086, m=23	1.23 [0.44 – 3.40]<br>n=47086, m=4	0.77 [0.28 – 2.14]<br>n=47086, m=4	Not calculated<br>n=47086, m=0
Both/mixed dairy and cow farming (individuals performing both dairy and cow farming)	0.98 [0.70 – 1.37]<br>n=30729, m=36	1.75 [1.09 – 2.81]<br>n=30729, m=19	1.62 [0.64 – 4.09]<br>n=30729, m=5	0.91 [0.37 – 2.24]<br>n=30729, m=5	2.67 [1.32 – 5.39]<br>n=30729, m=9	0.62 [0.34 – 1.13]<br>n=30729, m=11	Not calculated<br>n=30729, m=1	1.24 [0.50 – 3.10]<br>n=30729, m=5	Not calculated<br>n=30729, m=0
Cow farming (individuals performing only cow farming)	1.03 [0.85 – 1.25]<br>n=110214, m=121	1.30 [0.94 – 1.79]<br>n=110214, m=46	1.05 [0.51 – 2.14]<br>n=110214, m=9	1.69 [1.11 – 2.56]<br>n=110214, m=29	0.72 [0.36 – 1.45]<br>n=110214, m=9	0.78 [0.57 – 1.07]<br>n=110214, m=45	0.63 [0.29 – 1.39]<br>n=110214, m=7	1.84 [1.11 – 3.02]<br>n=110214, m=21	Not calculated<br>n=110214, m=2
Dairy farming (individuals performing only dairy farming)	1.04 [0.87 – 1.23]<br>n=158706, m=186	0.78 [0.56 – 1.10]<br>n=158706, m=45	1.25 [0.69 – 2.24]<br>n=158706, m=18	0.74 [0.45 – 1.21]<br>n=158706, m=20	0.62 [0.33 – 1.18]<br>n=158706, m=12	1.18 [0.92 – 1.51]<br>n=158706, m=99	1.53 [0.84 – 2.81]<br>n=158706, m=16	0.77 [0.43 – 1.37]<br>n=158706, m=16	2.10 [1.01 – 4.34]<br>n=158706, m=13
Unspecified specialized crop farming (e.g. horticulture)	1.21 [0.45 – 3.23]<br>n=6168, m=4	Not calculated<br>n=6168, m=2	Not calculated<br>n=6168, m=0	Not calculated<br>n=6168, m=2	Not calculated<br>n=6168, m=0	Not calculated<br>n=6168, m=1	Not calculated<br>n=6168, m=1	Not calculated<br>n=6168, m=0	Not calculated<br>n=6168, m=0
Sylviculture/forestry	Not calculated<br>n=1986, m=2	Not calculated<br>n=1986, m=0	Not calculated<br>n=1986, m=0	Not calculated<br>n=1986, m=0	Not calculated<br>n=1986, m=0	Not calculated<br>n=1986, m=2	Not calculated<br>n=1986, m=0	Not calculated<br>n=1986, m=0	Not calculated<br>n=1986, m=0
Viticulture	1.21 [0.98 – 1.48]<br>n=118577, m=113	1.31 [0.92 – 1.85]<br>n=118577, m=40	0.97 [0.41 – 2.33]<br>n=118577, m=6	1.59 [0.99 – 2.55]<br>n=118577, m=22	1.15 [0.62 – 2.11]<br>n=118577, m=13	1.29 [0.95 – 1.74]<br>n=118577, m=53	1.49 [0.78 – 2.84]<br>n=118577, m=12	0.70 [0.33 – 1.47]<br>n=118577, m=8	Not calculated<br>n=118577, m=1
Wheat and industrial grower (crop farming)	1.20 [1.03 – 1.41]<br>n=305838, m=258	1.13 [0.86 – 1.48]<br>n=305838, m=87	1.43 [0.80 – 2.55]<br>n=305838, m=19	1.18 [0.81 – 1.73]<br>n=305838, m=46	1.05 [0.66 – 1.67]<br>n=305838, m=29	1.28 [1.01 – 1.61]<br>n=305838, m=116	1.64 [1.00 – 2.69]<br>n=305838, m=29	1.04 [0.62 – 1.76]<br>n=305838, m=21	1.66 [0.77 – 3.59]<br>n=305838, m=11
Garden center	0.87 [0.28 – 2.70]<br>n=5111, m=3	Not calculated<br>n=5111, m=2	Not calculated<br>n=5111, m=0	Not calculated<br>n=5111, m=2	Not calculated<br>n=5111, m=0	Not calculated<br>n=5111, m=0	Not calculated<br>n=5111, m=1	Not calculated<br>n=5111, m=0	Not calculated<br>n=5111, m=0
Fruit arboriculture	1.50 [1.02 – 2.21]<br>n=24086, m=27	0.5 [0.16 – 1.56]<br>n=24086, m=3	Not calculated<br>n=24086, m=0	Not calculated<br>n=24086, m=2	Not calculated<br>n=24086, m=1	1.72 [1.00 – 2.94]<br>n=24086, m=14	2.11 [0.66 – 6.79]<br>n=24086, m=3	3.05 [1.31 – 7.08]<br>n=24086, m=6	Not calculated<br>n=24086, m=2
Truck farming, floriculture/flower–growing	1.36 [1.00 – 1.84]<br>n=41525, m=44	1.46 [0.85 – 2.51]<br>n=41525, m=14	1.79 [0.56 – 5.73]<br>n=41525, m=3	1.69 [0.82 – 3.48]<br>n=41525, m=8	0.87 [0.27 – 2.76]<br>n=41525, m=3	1.08 [0.66 – 1.76]<br>n=41525, m=17	1.11 [0.35 – 3.56]<br>n=41525, m=3	1.52 [0.66 – 3.51]<br>n=41525, m=6	3.63 [1.27 – 10.4]<br>n=41525, m=4

Fig. S3: Agricultural practices and risks of CNS tumors, TRACTOR, France, 2002-2016

Note: n: number of exposed farm managers, m: number of exposed cases, NC: not calculated, CNS: central nervous system, C70: malignant neoplasm of meninges, D32: benign neoplasm of meninges, D42: neoplasm of uncertain or unknown behavior of meninges, 95% CI: 95% confidence interval. Hazard ratios were estimated by Cox models with time to first CNS tumor insurance declaration as the underlying timescale, when the number of exposed cases was sufficient ($m \geq 3$), adjusted for sex, age, first year of the farm's establishment, farm surface, earnings, number of associates, unemployment status, total number of farms, family status, partner work status, farm location, number of comorbidities and having a secondary activity. Cells with red background refer to increased risks of CNS tumors.

	All CNS tumors	All meningiomas	C70	D32	D42	C71	D33	D43	C72
Rural craftsperson	Not calculated<br>n=256, m=0	Not calculated<br>n=256, m=0	Not calculated<br>n=256, m=0	Not calculated<br>n=256, m=0	Not calculated<br>n=256, m=0	Not calculated<br>n=256, m=0	Not calculated<br>n=256, m=0	Not calculated<br>n=256, m=0	Not calculated<br>n=256, m=0
Company representative/authorized representative	Not calculated<br>n=1435, m=2	Not calculated<br>n=1435, m=1	Not calculated<br>n=1435, m=0	Not calculated<br>n=1435, m=1	Not calculated<br>n=1435, m=0	Not calculated<br>n=1435, m=0	Not calculated<br>n=1435, m=0	Not calculated<br>n=1435, m=1	Not calculated<br>n=1435, m=0
Gardening, landscaping and reforestation companies	6.25 [2.91 – 13.4]<br>n=2369, m=7	5.94 [1.85 – 19.0]<br>n=2369, m=3	Not calculated<br>n=2369, m=2	Not calculated<br>n=2369, m=1	Not calculated<br>n=2369, m=0	Not calculated<br>n=2369, m=1	Not calculated<br>n=2369, m=1	Not calculated<br>n=2369, m=1	Not calculated<br>n=2369, m=1
Agricultural work companies	Not calculated<br>n=1715, m=0	Not calculated<br>n=1715, m=0	Not calculated<br>n=1715, m=0	Not calculated<br>n=1715, m=0	Not calculated<br>n=1715, m=0	Not calculated<br>n=1715, m=0	Not calculated<br>n=1715, m=0	Not calculated<br>n=1715, m=0	Not calculated<br>n=1715, m=0
Fixed sawmill	Not calculated<br>n=48, m=0	Not calculated<br>n=48, m=0	Not calculated<br>n=48, m=0	Not calculated<br>n=48, m=0	Not calculated<br>n=48, m=0	Not calculated<br>n=48, m=0	Not calculated<br>n=48, m=0	Not calculated<br>n=48, m=0	Not calculated<br>n=48, m=0
Wood production	Not calculated<br>n=283, m=0	Not calculated<br>n=283, m=0	Not calculated<br>n=283, m=0	Not calculated<br>n=283, m=0	Not calculated<br>n=283, m=0	Not calculated<br>n=283, m=0	Not calculated<br>n=283, m=0	Not calculated<br>n=283, m=0	Not calculated<br>n=283, m=0
Salt marsh	Not calculated<br>n=200, m=0	Not calculated<br>n=200, m=0	Not calculated<br>n=200, m=0	Not calculated<br>n=200, m=0	Not calculated<br>n=200, m=0	Not calculated<br>n=200, m=0	Not calculated<br>n=200, m=0	Not calculated<br>n=200, m=0	Not calculated<br>n=200, m=0
Shellfish farming	Not calculated<br>n=666, m=0	Not calculated<br>n=666, m=0	Not calculated<br>n=666, m=0	Not calculated<br>n=666, m=0	Not calculated<br>n=666, m=0	Not calculated<br>n=666, m=0	Not calculated<br>n=666, m=0	Not calculated<br>n=666, m=0	Not calculated<br>n=666, m=0
Unspecified and mixed farming (e.g. polyculture, mixed farming)	1.54 [1.16 – 2.05]<br>n=36955, m=59	1.28 [0.83 – 1.96]<br>n=36955, m=25	1.11 [0.39 – 3.16]<br>n=36955, m=4	0.82 [0.41 – 1.63]<br>n=36955, m=9	2.34 [1.26 – 4.35]<br>n=36955, m=14	1.34 [0.81 – 2.21]<br>n=36955, m=19	1.94 [0.73 – 5.15]<br>n=36955, m=5	2.27 [0.98 – 5.28]<br>n=36955, m=7	2.23 [0.79 – 6.26]<br>n=36955, m=5
Training, dressage, riding clubs	Not calculated<br>n=6049, m=2	Not calculated<br>n=6049, m=1	Not calculated<br>n=6049, m=1	Not calculated<br>n=6049, m=0	Not calculated<br>n=6049, m=0	Not calculated<br>n=6049, m=1	Not calculated<br>n=6049, m=0	Not calculated<br>n=6049, m=0	Not calculated<br>n=6049, m=0
Unspecified small animal farming (e.g. frogs, snails, bees)	1.88 [0.77 – 4.59]<br>n=7698, m=5	Not calculated<br>n=7698, m=1	Not calculated<br>n=7698, m=0	Not calculated<br>n=7698, m=2	Not calculated<br>n=7698, m=0	Not calculated<br>n=7698, m=1	Not calculated<br>n=7698, m=1	Not calculated<br>n=7698, m=1	Not calculated<br>n=7698, m=0
Poultry and rabbit farming	1.22 [0.71 – 2.11]<br>n=9671, m=14	0.70 [0.25 – 1.91]<br>n=9671, m=4	Not calculated<br>n=9671, m=1	Not calculated<br>n=9671, m=1	Not calculated<br>n=9671, m=2	2.31 [1.10 – 4.85]<br>n=9671, m=8	Not calculated<br>n=9671, m=1	Not calculated<br>n=9671, m=0	Not calculated<br>n=9671, m=2
Unspecified large animal farming (e.g. large dogs, zoo)	5.71 [1.82 – 17.9]<br>n=1280, m=3	Not calculated<br>n=1280, m=2	Not calculated<br>n=1280, m=1	Not calculated<br>n=1280, m=1	Not calculated<br>n=1280, m=0	Not calculated<br>n=1280, m=0	Not calculated<br>n=1280, m=0	Not calculated<br>n=1280, m=1	Not calculated<br>n=1280, m=0
Stud farming	1.14 [0.47 – 2.82]<br>n=6831, m=5	Not calculated<br>n=6831, m=2	Not calculated<br>n=6831, m=0	Not calculated<br>n=6831, m=2	Not calculated<br>n=6831, m=0	1.99 [0.61 – 6.52]<br>n=6831, m=3	Not calculated<br>n=6831, m=0	Not calculated<br>n=6831, m=0	Not calculated<br>n=6831, m=0
Pig farming	0.95 [0.38 – 2.33]<br>n=3830, m=5	Not calculated<br>n=3830, m=2	Not calculated<br>n=3830, m=0	Not calculated<br>n=3830, m=1	Not calculated<br>n=3830, m=1	1.46 [0.45 – 4.74]<br>n=3830, m=3	Not calculated<br>n=3830, m=0	Not calculated<br>n=3830, m=0	Not calculated<br>n=3830, m=0
Ovine and caprine farming	0.86 [0.52 – 1.44]<br>n=16808, m=16	0.99 [0.50 – 1.96]<br>n=16808, m=9	Not calculated<br>n=16808, m=1	0.76 [0.27 – 2.08]<br>n=16808, m=4	1.61 [0.57 – 4.57]<br>n=16808, m=4	0.63 [0.23 – 1.72]<br>n=16808, m=4	2.28 [0.67 – 7.74]<br>n=16808, m=3	Not calculated<br>n=16808, m=1	Not calculated<br>n=16808, m=0
Both/mixed dairy and cow farming (individuals performing both dairy and cow farming)	1.13 [0.67 – 1.91]<br>n=8004, m=15	1.06 [0.49 – 2.28]<br>n=8004, m=7	Not calculated<br>n=8004, m=2	Not calculated<br>n=8004, m=2	1.38 [0.42 – 4.50]<br>n=8004, m=3	1.01 [0.41 – 2.49]<br>n=8004, m=5	Not calculated<br>n=8004, m=0	2.81 [0.83 – 9.51]<br>n=8004, m=3	Not calculated<br>n=8004, m=0
Cow farming (individuals performing only cow farming)	1.11 [0.82 – 1.50]<br>n=32699, m=50	1.34 [0.90 – 2.00]<br>n=32699, m=30	1.11 [0.43 – 2.89]<br>n=32699, m=5	1.68 [1.01 – 2.79]<br>n=32699, m=20	0.83 [0.35 – 1.97]<br>n=32699, m=6	0.59 [0.31 – 1.14]<br>n=32699, m=10	0.78 [0.23 – 2.61]<br>n=32699, m=3	2.25 [0.96 – 5.28]<br>n=32699, m=7	Not calculated<br>n=32699, m=0
Dairy farming (individuals performing only dairy farming)	0.77 [0.57 – 1.03]<br>n=48823, m=62	0.69 [0.44 – 1.07]<br>n=48823, m=26	1.29 [0.58 – 2.89]<br>n=48823, m=10	0.52 [0.26 – 1.04]<br>n=48823, m=10	0.79 [0.38 – 1.67]<br>n=48823, m=10	0.57 [0.33 – 0.98]<br>n=48823, m=18	2.01 [0.81 – 4.97]<br>n=48823, m=8	0.66 [0.25 – 1.72]<br>n=48823, m=6	2.77 [1.04 – 7.38]<br>n=48823, m=6
Unspecified specialized crop farming (e.g. horticulture)	Not calculated<br>n=2231, m=2	Not calculated<br>n=2231, m=1	Not calculated<br>n=2231, m=0	Not calculated<br>n=2231, m=1	Not calculated<br>n=2231, m=0	Not calculated<br>n=2231, m=1	Not calculated<br>n=2231, m=0	Not calculated<br>n=2231, m=0	Not calculated<br>n=2231, m=0
Sylviculture/forestry	Not calculated<br>n=339, m=0	Not calculated<br>n=339, m=0	Not calculated<br>n=339, m=0	Not calculated<br>n=339, m=0	Not calculated<br>n=339, m=0	Not calculated<br>n=339, m=0	Not calculated<br>n=339, m=0	Not calculated<br>n=339, m=0	Not calculated<br>n=339, m=0
Viticulture	1.29 [0.94 – 1.77]<br>n=41970, m=49	1.48 [0.96 – 2.28]<br>n=41970, m=27	2.12 [0.83 – 5.43]<br>n=41970, m=6	1.54 [0.86 – 2.75]<br>n=41970, m=15	1.02 [0.44 – 2.36]<br>n=41970, m=7	1.57 [0.91 – 2.71]<br>n=41970, m=17	0.83 [0.24 – 2.87]<br>n=41970, m=3	Not calculated<br>n=41970, m=2	Not calculated<br>n=41970, m=0
Wheat and industrial grower (crop farming)	1.11 [0.87 – 1.41]<br>n=10224, m=113	1.17 [0.82 – 1.66]<br>n=10224, m=55	1.04 [0.48 – 2.27]<br>n=10224, m=11	1.24 [0.77 – 2.00]<br>n=10224, m=32	1.13 [0.60 – 2.12]<br>n=10224, m=17	1.12 [0.72 – 1.74]<br>n=10224, m=36	1.19 [0.53 – 2.64]<br>n=10224, m=11	0.99 [0.44 – 2.22]<br>n=10224, m=10	1.10 [0.36 – 3.35]<br>n=10224, m=4
Garden center	Not calculated<br>n=1358, m=2	Not calculated<br>n=1358, m=2	Not calculated<br>n=1358, m=0	Not calculated<br>n=1358, m=2	Not calculated<br>n=1358, m=0	Not calculated<br>n=1358, m=0	Not calculated<br>n=1358, m=0	Not calculated<br>n=1358, m=0	Not calculated<br>n=1358, m=0
Fruit arboriculture	1.25 [0.66 – 2.35]<br>n=7649, m=10	Not calculated<br>n=7649, m=2	Not calculated<br>n=7649, m=0	Not calculated<br>n=7649, m=1	Not calculated<br>n=7649, m=1	2.58 [1.12 – 5.96]<br>n=7649, m=6	Not calculated<br>n=7649, m=1	Not calculated<br>n=7649, m=1	Not calculated<br>n=7649, m=0
Truck farming, floriculture/flower–growing	1.40 [0.86 – 2.29]<br>n=12672, m=17	1.45 [0.71 – 2.97]<br>n=12672, m=8	Not calculated<br>n=12672, m=2	1.32 [0.48 – 3.64]<br>n=12672, m=4	Not calculated<br>n=12672, m=2	1.23 [0.50 – 3.03]<br>n=12672, m=5	Not calculated<br>n=12672, m=1	Not calculated<br>n=12672, m=2	Not calculated<br>n=12672, m=1

Fig.S4: Agricultural practices and risks of CNS tumors for women, TRACTOR, France, 2002-2016

Note: n: number of exposed farm managers, m: number of exposed cases, NC: not calculated, CNS: central nervous system, C70: malignant neoplasm of meninges, D32: benign neoplasm of meninges, D42: neoplasm of uncertain or unknown behavior of meninges, 95% CI: 95% confidence interval.

Hazard ratios were estimated by Cox models with time to first CNS tumor insurance declaration as the underlying timescale, when the number of exposed cases was sufficient ($m \geq 3$), adjusted for age, first year of the farm's establishment, farm surface, earnings, number of associates, unemployment status, total number of farms, family status, partner work status, farm location, number of comorbidities and having a secondary activity.

Cells with red background refer to increased risks of CNS tumors, while cells with green background refer to decreased risk of CNS tumors.

	All CNS tumors	All meningiomas	C70	D32	D42	C71	D33	D43	C72
Rural craftsperson	Not calculated<br>n=6782, m=0	Not calculated<br>n=6782, m=0	Not calculated<br>n=6782, m=0	Not calculated<br>n=6782, m=0	Not calculated<br>n=6782, m=0	Not calculated<br>n=6782, m=0	Not calculated<br>n=6782, m=0	Not calculated<br>n=6782, m=0	Not calculated<br>n=6782, m=0
Company representative/authorized representative	Not calculated<br>n=408, m=1	Not calculated<br>n=408, m=0	Not calculated<br>n=408, m=0	Not calculated<br>n=408, m=0	Not calculated<br>n=408, m=0	Not calculated<br>n=408, m=1	Not calculated<br>n=408, m=0	Not calculated<br>n=408, m=0	Not calculated<br>n=408, m=0
Gardening, landscaping and reforestation companies	0.86 [0.55 – 1.34]<br>n=42579, m=21	Not calculated<br>n=42579, m=2	Not calculated<br>n=42579, m=1	Not calculated<br>n=42579, m=1	Not calculated<br>n=42579, m=0	0.88 [0.49 – 1.58]<br>n=42579, m=12	1.33 [0.39 – 4.52]<br>n=42579, m=3	1.02 [0.35 – 2.93]<br>n=42579, m=4	Not calculated<br>n=42579, m=0
Agricultural work companies	1.41 [0.77 – 2.56]<br>n=12567, m=11	2.69 [0.99 – 7.35]<br>n=12567, m=4	Not calculated<br>n=12567, m=0	Not calculated<br>n=12567, m=1	5.46 [1.66 – 17.9]<br>n=12567, m=3	0.91 [0.34 – 2.45]<br>n=12567, m=4	Not calculated<br>n=12567, m=2	Not calculated<br>n=12567, m=1	Not calculated<br>n=12567, m=0
Fixed sawmill	Not calculated<br>n=687, m=0	Not calculated<br>n=687, m=0	Not calculated<br>n=687, m=0	Not calculated<br>n=687, m=0	Not calculated<br>n=687, m=0	Not calculated<br>n=687, m=0	Not calculated<br>n=687, m=0	Not calculated<br>n=687, m=0	Not calculated<br>n=687, m=0
Wood production	1.07 [0.57 – 2.01]<br>n=10187, m=10	Not calculated<br>n=10187, m=2	Not calculated<br>n=10187, m=0	Not calculated<br>n=10187, m=1	Not calculated<br>n=10187, m=1	1.25 [0.56 – 2.83]<br>n=10187, m=6	Not calculated<br>n=10187, m=0	Not calculated<br>n=10187, m=1	Not calculated<br>n=10187, m=1
Salt marsh	Not calculated<br>n=673, m=0	Not calculated<br>n=673, m=0	Not calculated<br>n=673, m=0	Not calculated<br>n=673, m=0	Not calculated<br>n=673, m=0	Not calculated<br>n=673, m=0	Not calculated<br>n=673, m=0	Not calculated<br>n=673, m=0	Not calculated<br>n=673, m=0
Shellfish farming	Not calculated<br>n=2684, m=2	Not calculated<br>n=2684, m=0	Not calculated<br>n=2684, m=0	Not calculated<br>n=2684, m=0	Not calculated<br>n=2684, m=0	Not calculated<br>n=2684, m=2	Not calculated<br>n=2684, m=0	Not calculated<br>n=2684, m=0	Not calculated<br>n=2684, m=0
Unspecified and mixed farming (e.g. polyculture, mixed farming)	1.21 [0.96 – 1.54]<br>n=83791, m=81	0.77 [0.41 – 1.44]<br>n=83791, m=11	0.84 [0.25 – 2.83]<br>n=83791, m=3	0.47 [0.15 – 1.53]<br>n=83791, m=3	1.09 [0.45 – 2.64]<br>n=83791, m=6	1.47 [1.09 – 1.98]<br>n=83791, m=53	1.24 [0.57 – 2.68]<br>n=83791, m=8	1.23 [0.62 – 2.43]<br>n=83791, m=10	Not calculated<br>n=83791, m=1
Training, dressage, riding clubs	1.34 [0.60 – 3.02]<br>n=7224, m=6	Not calculated<br>n=7224, m=1	Not calculated<br>n=7224, m=0	Not calculated<br>n=7224, m=0	Not calculated<br>n=7224, m=1	1.16 [0.37 – 3.64]<br>n=7224, m=3	Not calculated<br>n=7224, m=0	Not calculated<br>n=7224, m=2	Not calculated<br>n=7224, m=0
Unspecified small animal farming (e.g. frogs, snails, bees)	1.28 [0.53 – 3.09]<br>n=10360, m=5	Not calculated<br>n=10360, m=1	Not calculated<br>n=10360, m=0	Not calculated<br>n=10360, m=0	Not calculated<br>n=10360, m=1	1.37 [0.44 – 4.28]<br>n=10360, m=3	Not calculated<br>n=10360, m=0	Not calculated<br>n=10360, m=1	Not calculated<br>n=10360, m=0
Poultry and rabbit farming	0.92 [0.51 – 1.68]<br>n=14905, m=11	Not calculated<br>n=14905, m=2	Not calculated<br>n=14905, m=0	Not calculated<br>n=14905, m=1	Not calculated<br>n=14905, m=1	0.66 [0.27 – 1.61]<br>n=14905, m=5	Not calculated<br>n=14905, m=2	Not calculated<br>n=14905, m=2	Not calculated<br>n=14905, m=0
Unspecified large animal farming (e.g. large dogs, zoo)	Not calculated<br>n=1383, m=1	Not calculated<br>n=1383, m=0	Not calculated<br>n=1383, m=0	Not calculated<br>n=1383, m=0	Not calculated<br>n=1383, m=0	Not calculated<br>n=1383, m=1	Not calculated<br>n=1383, m=0	Not calculated<br>n=1383, m=0	Not calculated<br>n=1383, m=0
Stud farming	Not calculated<br>n=8810, m=1	Not calculated<br>n=8810, m=1	Not calculated<br>n=8810, m=0	Not calculated<br>n=8810, m=1	Not calculated<br>n=8810, m=0	Not calculated<br>n=8810, m=0	Not calculated<br>n=8810, m=0	Not calculated<br>n=8810, m=0	Not calculated<br>n=8810, m=0
Pig farming	2.18 [1.35 – 3.52]<br>n=9559, m=19	2.53 [0.88 – 7.24]<br>n=9559, m=4	Not calculated<br>n=9559, m=1	Not calculated<br>n=9559, m=0	8.11 [2.29 – 28.7]<br>n=9559, m=3	2.52 [1.43 – 4.45]<br>n=9559, m=14	Not calculated<br>n=9559, m=0	Not calculated<br>n=9559, m=0	Not calculated<br>n=9559, m=1
Ovine and caprine farming	1.33 [0.91 – 1.94]<br>n=30278, m=29	1.95 [0.89 – 4.25]<br>n=30278, m=7	Not calculated<br>n=30278, m=1	Not calculated<br>n=30278, m=2	2.50 [0.87 – 7.20]<br>n=30278, m=4	1.55 [0.96 – 2.49]<br>n=30278, m=19	Not calculated<br>n=30278, m=1	0.89 [0.27 – 2.91]<br>n=30278, m=3	Not calculated<br>n=30278, m=0
Both/mixed dairy and cow farming (individuals performing both dairy and cow farming)	0.89 [0.57 – 1.39]<br>n=22725, m=21	2.69 [1.46 – 4.97]<br>n=22725, m=12	2.14 [0.63 – 7.26]<br>n=22725, m=3	1.33 [0.41 – 4.36]<br>n=22725, m=3	4.83 [1.95 – 12.0]<br>n=22725, m=6	0.43 [0.19 – 0.97]<br>n=22725, m=6	Not calculated<br>n=22725, m=1	Not calculated<br>n=22725, m=2	Not calculated<br>n=22725, m=0
Cow farming (individuals performing only cow farming)	0.97 [0.75 – 1.25]<br>n=77515, m=71	1.17 [0.68 – 2.01]<br>n=77515, m=16	1.12 [0.38 – 3.31]<br>n=77515, m=4	1.58 [0.75 – 3.31]<br>n=77515, m=9	0.51 [0.16 – 1.66]<br>n=77515, m=3	0.82 [0.57 – 1.17]<br>n=77515, m=35	0.56 [0.20 – 1.58]<br>n=77515, m=4	1.78 [0.96 – 3.28]<br>n=77515, m=14	Not calculated<br>n=77515, m=2
Dairy farming (individuals performing only dairy farming)	1.22 [0.98 – 1.52]<br>n=109883, m=124	0.80 [0.48 – 1.36]<br>n=109883, m=19	1.22 [0.51 – 2.95]<br>n=109883, m=8	1.00 [0.48 – 2.09]<br>n=109883, m=10	Not calculated<br>n=109883, m=2	1.43 [1.08 – 1.89]<br>n=109883, m=81	1.38 [0.61 – 3.13]<br>n=109883, m=8	0.83 [0.40 – 1.72]<br>n=109883, m=10	1.67 [0.63 – 4.43]<br>n=109883, m=7
Unspecified specialized crop farming (e.g. horticulture)	Not calculated<br>n=3933, m=2	Not calculated<br>n=3933, m=1	Not calculated<br>n=3933, m=0	Not calculated<br>n=3933, m=1	Not calculated<br>n=3933, m=0	Not calculated<br>n=3933, m=0	Not calculated<br>n=3933, m=1	Not calculated<br>n=3933, m=0	Not calculated<br>n=3933, m=0
Sylviculture/forestry	Not calculated<br>n=1647, m=2	Not calculated<br>n=1647, m=0	Not calculated<br>n=1647, m=0	Not calculated<br>n=1647, m=0	Not calculated<br>n=1647, m=0	Not calculated<br>n=1647, m=2	Not calculated<br>n=1647, m=0	Not calculated<br>n=1647, m=0	Not calculated<br>n=1647, m=0
Viticulture	1.14 [0.87 – 1.49]<br>n=76607, m=64	1.08 [0.60 – 1.97]<br>n=76607, m=13	Not calculated<br>n=76607, m=0	1.76 [0.76 – 4.04]<br>n=76607, m=7	1.19 [0.48 – 2.97]<br>n=76607, m=6	1.16 [0.81 – 1.67]<br>n=76607, m=36	2.00 [0.94 – 4.29]<br>n=76607, m=9	0.87 [0.37 – 2.06]<br>n=76607, m=6	Not calculated<br>n=76607, m=1
Wheat and industrial grower (crop farming)	1.21 [0.99 – 1.47]<br>n=203598, m=145	1.18 [0.77 – 1.83]<br>n=203598, m=32	1.80 [0.75 – 4.36]<br>n=203598, m=8	1.21 [0.62 – 2.36]<br>n=203598, m=14	1.03 [0.51 – 2.10]<br>n=203598, m=12	1.22 [0.93 – 1.59]<br>n=203598, m=80	1.88 [1.01 – 3.50]<br>n=203598, m=18	0.76 [0.38 – 1.53]<br>n=203598, m=11	2.32 [0.90 – 5.95]<br>n=203598, m=7
Garden center	Not calculated<br>n=3753, m=1	Not calculated<br>n=3753, m=0	Not calculated<br>n=3753, m=0	Not calculated<br>n=3753, m=0	Not calculated<br>n=3753, m=0	Not calculated<br>n=3753, m=0	Not calculated<br>n=3753, m=1	Not calculated<br>n=3753, m=0	Not calculated<br>n=3753, m=0
Fruit arboriculture	1.67 [1.03 – 2.72]<br>n=16437, m=17	Not calculated<br>n=16437, m=1	Not calculated<br>n=16437, m=0	Not calculated<br>n=16437, m=1	Not calculated<br>n=16437, m=0	1.36 [0.67 – 2.76]<br>n=16437, m=8	Not calculated<br>n=16437, m=2	4.02 [1.57 – 10.3]<br>n=16437, m=5	Not calculated<br>n=16437, m=2
Truck farming, floriculture/flower–growing	1.36 [0.92 – 2.01]<br>n=28853, m=27	1.58 [0.69 – 3.62]<br>n=28853, m=6	Not calculated<br>n=28853, m=1	2.40 [0.85 – 6.77]<br>n=28853, m=4	Not calculated<br>n=28853, m=1	1.02 [0.57 – 1.82]<br>n=28853, m=12	Not calculated<br>n=28853, m=2	1.58 [0.57 – 4.38]<br>n=28853, m=4	5.42 [1.55 – 18.9]<br>n=28853, m=3

Fig.S5: Agricultural practices and risks of CNS tumors for men, TRACTOR, France, 2002-2016

Note: n: number of exposed farm managers, m: number of exposed cases, NC: not calculated, CNS: central nervous system, C70: malignant neoplasm of meninges, D32: benign neoplasm of meninges, D42: neoplasm of uncertain or unknown behavior of meninges, 95% CI: 95% confidence interval.

Hazard ratios were estimated by Cox models with time to first CNS tumor insurance declaration as the underlying timescale, when the number of exposed cases was sufficient ($m \geq 3$), adjusted for age, first year of the farm's establishment, farm surface, earnings, number of associates, unemployment status, total number of farms, family status, partner work status, farm location, number of comorbidities and having a secondary activity.

Cells with red background refer to increased risks of CNS tumors, while cells with green background refer to decreased risk of CNS tumors.

	All CNS tumors	All meningiomas	C70	D32	D42	C71	D33	D43	C72
Rural craftsperson	Not calculated<br>n=7038, m=0	Not calculated<br>n=7038, m=0	Not calculated<br>n=7038, m=0	Not calculated<br>n=7038, m=0	Not calculated<br>n=7038, m=0	Not calculated<br>n=7038, m=0	Not calculated<br>n=7038, m=0	Not calculated<br>n=7038, m=0	Not calculated<br>n=7038, m=0
Company representative/authorized representative	3.09 [0.99−9.63]<br>n=1843, m=3	Not calculated<br>n=1845, m=1	Not calculated<br>n=1846, m=0	Not calculated<br>n=1845, m=1	Not calculated<br>n=1846, m=0	Not calculated<br>n=1846, m=0	Not calculated<br>n=1845, m=1	Not calculated<br>n=1846, m=0	Not calculated<br>n=1845, m=1	Not calculated<br>n=1846, m=0
Gardening, landscaping and reforestation companies	1.07 [0.67−1.7]<br>n=44920, m=28	0.77 [0.28−2.11]<br>n=44943, m=5	2.86 [0.87−9.42]<br>n=44945, m=3	Not calculated<br>n=44946, m=2	Not calculated<br>n=44948, m=0	1.06 [0.53−2.11]<br>n=44935, m=13	1.36 [0.48−3.82]<br>n=44944, m=4	1.18 [0.37−3.76]<br>n=44943, m=5	Not calculated<br>n=44947, m=1
Agricultural work companies	1.21 [0.65−2.21]<br>n=14271, m=11	1.83 [0.66−5.08]<br>n=14278, m=4	Not calculated<br>n=14282, m=0	Not calculated<br>n=14281, m=1	3.74 [1.17−12]<br>n=14279, m=3	0.77 [0.28−2.08]<br>n=14278, m=4	Not calculated<br>n=14280, m=2	Not calculated<br>n=14281, m=1	Not calculated<br>n=14282, m=0
Fixed sawmill	Not calculated<br>n=735, m=0	Not calculated<br>n=735, m=0	Not calculated<br>n=735, m=0	Not calculated<br>n=735, m=0	Not calculated<br>n=735, m=0	Not calculated<br>n=735, m=0	Not calculated<br>n=735, m=0	Not calculated<br>n=735, m=0	Not calculated<br>n=735, m=0
Wood production	1.13 [0.60−2.12]<br>n=10460, m=10	Not calculated<br>n=10468, m=2	Not calculated<br>n=10470, m=0	Not calculated<br>n=10469, m=1	Not calculated<br>n=10469, m=1	1.68 [0.70−4.01]<br>n=10464, m=6	Not calculated<br>n=10470, m=0	Not calculated<br>n=10469, m=1	Not calculated<br>n=10469, m=1
Salt marsh	Not calculated<br>n=873, m=0	Not calculated<br>n=873, m=0	Not calculated<br>n=873, m=0	Not calculated<br>n=873, m=0	Not calculated<br>n=873, m=0	Not calculated<br>n=873, m=0	Not calculated<br>n=873, m=0	Not calculated<br>n=873, m=0	Not calculated<br>n=873, m=0
Unspecified and mixed farming (e.g. polyculture, mixed farming)	1.24 [1.01−1.53]<br>n=120606, m=140	1.01 [0.68−1.49]<br>n=120710, m=36	0.69 [0.28−1.7]<br>n=120739, m=7	0.59 [0.30−1.13]<br>n=120734, m=12	2.03 [1.16−3.54]<br>n=120726, m=20	1.29 [0.96−1.74]<br>n=120674, m=72	1.16 [0.57−2.33]<br>n=120733, m=13	2 [1.12−3.59]<br>n=120729, m=17	1.1 [0.39−3.02]<br>n=120740, m=6
Shellfish farming	Not calculated<br>n=3348, m=2	Not calculated<br>n=3350, m=0	Not calculated<br>n=3350, m=0	Not calculated<br>n=3350, m=0	Not calculated<br>n=3350, m=0	Not calculated<br>n=3348, m=2	Not calculated<br>n=3350, m=0	Not calculated<br>n=3350, m=0	Not calculated<br>n=3350, m=0
Training, dressage, riding clubs	0.67 [0.33−1.36]<br>n=13265, m=8	Not calculated<br>n=13271, m=2	Not calculated<br>n=13272, m=1	Not calculated<br>n=13273, m=0	Not calculated<br>n=13272, m=1	0.76 [0.28−2.04]<br>n=13269, m=4	Not calculated<br>n=13273, m=0	Not calculated<br>n=13271, m=2	Not calculated<br>n=13273, m=0
Unspecified small animal farming (e.g. frogs, snails, bees)	1.16 [0.62−2.16]<br>n=18048, m=10	1.11 [0.34−3.6]<br>n=18055, m=3	Not calculated<br>n=18058, m=0	Not calculated<br>n=18056, m=2	Not calculated<br>n=18057, m=1	1.04 [0.38−2.77]<br>n=18054, m=4	Not calculated<br>n=18057, m=1	Not calculated<br>n=18056, m=2	Not calculated<br>n=18058, m=0
Poultry and rabbit farming	0.92 [0.62−1.38]<br>n=24551, m=25	0.66 [0.29−1.49]<br>n=24570, m=6	Not calculated<br>n=24575, m=1	Not calculated<br>n=24574, m=2	0.96 [0.30−3.08]<br>n=24573, m=3	0.99 [0.57−1.73]<br>n=24563, m=13	1.43 [0.44−4.59]<br>n=24573, m=3	Not calculated<br>n=24574, m=2	Not calculated<br>n=24574, m=2
Unspecified large animal farming (e.g. large dogs, zoo)	2.3 [0.86−6.15]<br>n=2659, m=4	Not calculated<br>n=2661, m=2	Not calculated<br>n=2662, m=1	Not calculated<br>n=2662, m=1	Not calculated<br>n=2663, m=0	Not calculated<br>n=2662, m=1	Not calculated<br>n=2663, m=0	Not calculated<br>n=2662, m=1	Not calculated<br>n=2663, m=0
Stud farming	0.65 [0.29−1.46]<br>n=15635, m=6	0.95 [0.30−3]<br>n=15638, m=3	Not calculated<br>n=15641, m=0	1.89 [0.59−6.01]<br>n=15638, m=3	Not calculated<br>n=15641, m=0	0.69 [0.22−2.19]<br>n=15638, m=3	Not calculated<br>n=15641, m=0	Not calculated<br>n=15641, m=0	Not calculated<br>n=15641, m=0
Pig farming	1.55 [1.03−2.32]<br>n=13365, m=24	1.41 [0.61−3.24]<br>n=13383, m=6	Not calculated<br>n=13388, m=1	Not calculated<br>n=13388, m=1	2.72 [0.95−7.76]<br>n=13385, m=4	2.06 [1.24−3.41]<br>n=13372, m=17	Not calculated<br>n=13389, m=0	Not calculated<br>n=13389, m=0	Not calculated<br>n=13388, m=1
Ovine and caprine farming	0.98 [0.72−1.33]<br>n=47041, m=45	1.08 [0.65−1.8]<br>n=47070, m=16	Not calculated<br>n=47084, m=2	0.84 [0.37−1.93]<br>n=47080, m=6	1.66 [0.79−3.46]<br>n=47078, m=8	1.08 [0.70−1.64]<br>n=47063, m=23	1.07 [0.38−2.94]<br>n=47082, m=4	0.75 [0.27−2.06]<br>n=47082, m=4	Not calculated<br>n=47086, m=0
Both/mixed dairy and cow farming (individuals performing both dairy and cow farming)	0.89 [0.64−1.26]<br>n=30693, m=36	1.56 [0.97−2.5]<br>n=30710, m=19	1.51 [0.6−3.8]<br>n=30724, m=5	0.81 [0.33−2]<br>n=30724, m=5	2.51 [1.25−5.05]<br>n=30720, m=9	0.55 [0.30−1.01]<br>n=30718, m=11	Not calculated<br>n=30728, m=1	1.32 [0.52−3.27]<br>n=30724, m=5	Not calculated<br>n=30729, m=0
Cow farming (individuals performing only cow farming)	0.94 [0.77−1.15]<br>n=110093, m=121	1.16 [0.83−1.6]<br>n=110168, m=46	1.08 [0.53−2.16]<br>n=110204, m=10	1.6 [1.04−2.45]<br>n=110185, m=29	0.62 [0.31−1.27]<br>n=110205, m=9	0.69 [0.50−0.95]<br>n=110169, m=45	0.61 [0.27−1.35]<br>n=110207, m=7	2.02 [1.21−3.39]<br>n=110193, m=21	Not calculated<br>n=110212, m=2
Dairy farming (individuals performing only dairy farming)	0.95 [0.80−1.12]<br>n=158520, m=186	0.69 [0.49−0.95]<br>n=158661, m=45	1.11 [0.63−1.97]<br>n=158688, m=18	0.62 [0.38−1.02]<br>n=158686, m=20	0.58 [0.31−1.08]<br>n=158694, m=12	1.08 [0.85−1.37]<br>n=158607, m=99	1.16 [0.65−2.05]<br>n=158690, m=16	0.73 [0.42−1.29]<br>n=158690, m=16	2.08 [1.03−4.19]<br>n=158693, m=13
Unspecified specialized farming (e.g. horticulture)	1.04 [0.38−2.78]<br>n=6164, m=4	Not calculated<br>n=6166, m=2	Not calculated<br>n=6168, m=0	Not calculated<br>n=6166, m=2	Not calculated<br>n=6168, m=0	Not calculated<br>n=6167, m=1	Not calculated<br>n=6167, m=1	Not calculated<br>n=6168, m=0	Not calculated<br>n=6168, m=0
Sylviculture/forestry	Not calculated<br>n=1984, m=2	Not calculated<br>n=1986, m=0	Not calculated<br>n=1986, m=0	Not calculated<br>n=1986, m=0	Not calculated<br>n=1986, m=0	Not calculated<br>n=1984, m=2	Not calculated<br>n=1986, m=0	Not calculated<br>n=1986, m=0	Not calculated<br>n=1986, m=0
Viticulture	1.12 [0.88−1.4]<br>n=118464, m=113	1.41 [0.95−2.09]<br>n=118537, m=40	0.73 [0.28−1.89]<br>n=118571, m=6	1.63 [0.95−2.79]<br>n=118555, m=22	1.25 [0.63−2.49]<br>n=118564, m=13	1.09 [0.78−1.53]<br>n=118524, m=53	1.2 [0.58−2.45]<br>n=118565, m=12	0.76 [0.34−1.7]<br>n=118569, m=8	Not calculated<br>n=118576, m=1
Wheat and industrial grower (crop farming)	1.58 [1.35−1.86]<br>n=305580, m=258	1.67 [1.25−2.22]<br>n=305751, m=87	1.55 [0.84−2.83]<br>n=305819, m=19	1.98 [1.32−2.96]<br>n=305792, m=46	1.45 [0.88−2.37]<br>n=305809, m=29	1.52 [1.2−1.93]<br>n=305722, m=116	2.54 [1.5−4.33]<br>n=305809, m=29	1.1 [0.63−1.88]<br>n=305817, m=21	1.53 [0.69−3.35]<br>n=305827, m=11
Garden center	0.77 [0.24−2.4]<br>n=5108, m=3	Not calculated<br>n=5109, m=2	Not calculated<br>n=5111, m=0	Not calculated<br>n=5109, m=2	Not calculated<br>n=5111, m=0	Not calculated<br>n=5111, m=0	Not calculated<br>n=5110, m=1	Not calculated<br>n=5111, m=0	Not calculated<br>n=5111, m=0
Fruit arboriculture	1.5 [1.02−2.22]<br>n=24059, m=27	0.53 [0.17−1.67]<br>n=24083, m=3	Not calculated<br>n=24086, m=0	Not calculated<br>n=24084, m=2	Not calculated<br>n=24085, m=1	1.61 [0.93−2.77]<br>n=24072, m=14	1.85 [0.56−5.98]<br>n=24083, m=3	3.38 [1.46−7.83]<br>n=24080, m=6	Not calculated<br>n=24084, m=2
Truck farming, floriculture/flower−growing	1.45 [1.06−1.98]<br>n=41481, m=44	1.69 [0.97−2.94]<br>n=41511, m=14	1.86 [0.56−6.13]<br>n=41522, m=3	1.98 [0.94−4.16]<br>n=41517, m=8	0.96 [0.29−3.11]<br>n=41522, m=3	1.06 [0.64−1.76]<br>n=41508, m=17	1.05 [0.32−3.45]<br>n=41522, m=3	1.9 [0.80−4.48]<br>n=41519, m=6	3.91 [1.31−11.6]<br>n=41521, m=4

Fig.S6: Results from sensitivity analysis (age as continuous variable) - Agricultural practices and risks of CNS tumors for both sexes combined, TRACTOR, France, 2002-2016

Note: n: number of exposed farm managers, m: number of exposed cases, NC: not calculated, CNS: central nervous system, C70: malignant neoplasm of meninges, D32: benign neoplasm of meninges, D42: neoplasm of uncertain or unknown behavior of meninges, 95% CI: 95% confidence interval.
Hazard ratios were estimated by Cox models with time to first CNS tumor insurance declaration as the underlying timescale, when the number of exposed cases was sufficient (m ≥ 3), adjusted for sex, age, first year of the farm’s establishment, farm surface, earnings, number of associates, unemployment status, total number of farms, family status, partner work status, farm location, number of comorbidities and having a secondary activity.
Cells with red background refer to increased risks of CNS tumors, while cells with green background refer to decreased risk of CNS tumors.

	All CNS tumors	All meningiomas	C70	D32	D42	C71	D33	D43	C72
Rural craftsperson	Not calculated n=256, m=0	Not calculated n=256, m=0	Not calculated n=256, m=0	Not calculated n=256, m=0	Not calculated n=256, m=0	Not calculated n=256, m=0	Not calculated n=256, m=0	Not calculated n=256, m=0	Not calculated n=256, m=0
Company representative/authorized representative	Not calculated n=1435, m=2	Not calculated n=1436, m=1	Not calculated n=1437, m=0	Not calculated n=1436, m=1	Not calculated n=1437, m=0	Not calculated n=1437, m=0	Not calculated n=1437, m=0	Not calculated n=1436, m=1	Not calculated n=1437, m=0
Gardening, landscaping and reforestation companies	3.42 [1.61−7.27] n=2362, m=7	3.35 [1.06−10.6] n=2366, m=3	Not calculated n=2367, m=2	Not calculated n=2368, m=1	Not calculated n=2369, m=0	Not calculated n=2368, m=1	Not calculated n=2368, m=1	Not calculated n=2368, m=1	Not calculated n=2368, m=1
Agricultural work companies	Not calculated n=1715, m=0	Not calculated n=1715, m=0	Not calculated n=1715, m=0	Not calculated n=1715, m=0	Not calculated n=1715, m=0	Not calculated n=1715, m=0	Not calculated n=1715, m=0	Not calculated n=1715, m=0	Not calculated n=1715, m=0
Fixed sawmill	Not calculated n=48, m=0	Not calculated n=48, m=0	Not calculated n=48, m=0	Not calculated n=48, m=0	Not calculated n=48, m=0	Not calculated n=48, m=0	Not calculated n=48, m=0	Not calculated n=48, m=0	Not calculated n=48, m=0
Wood production	Not calculated n=283, m=0	Not calculated n=283, m=0	Not calculated n=283, m=0	Not calculated n=283, m=0	Not calculated n=283, m=0	Not calculated n=283, m=0	Not calculated n=283, m=0	Not calculated n=283, m=0	Not calculated n=283, m=0
Salt marsh	Not calculated n=200, m=0	Not calculated n=200, m=0	Not calculated n=200, m=0	Not calculated n=200, m=0	Not calculated n=200, m=0	Not calculated n=200, m=0	Not calculated n=200, m=0	Not calculated n=200, m=0	Not calculated n=200, m=0
Unspecified and mixed farming (e.g. polyculture, mixed farming)	1.48 [1.08−2.03] n=36896, m=59	1.26 [0.78−2.02] n=36930, m=25	0.70 [0.22−2.25] n=36951, m=4	0.69 [0.32−1.47] n=36946, m=9	2.74 [1.39−5.41] n=36941, m=14	1.29 [0.72−2.31] n=36936, m=19	1.62 [0.54−4.78] n=36950, m=5	2.7 [1.09−6.7] n=36948, m=7	3.14 [0.91−10.8] n=36950, m=5
Shellfish farming	Not calculated n=666, m=0	Not calculated n=666, m=0	Not calculated n=666, m=0	Not calculated n=666, m=0	Not calculated n=666, m=0	Not calculated n=666, m=0	Not calculated n=666, m=0	Not calculated n=666, m=0	Not calculated n=666, m=0
Training, dressage, riding clubs	Not calculated n=6047, m=2	Not calculated n=6048, m=1	Not calculated n=6048, m=1	Not calculated n=6049, m=0	Not calculated n=6049, m=0	Not calculated n=6048, m=1	Not calculated n=6049, m=0	Not calculated n=6049, m=0	Not calculated n=6049, m=0
Unspecified small animal farming (e.g. frogs, snails, bees)	1.21 [0.49−2.93] n=7693, m=5	Not calculated n=7696, m=2	Not calculated n=7698, m=0	Not calculated n=7696, m=2	Not calculated n=7698, m=0	Not calculated n=7697, m=1	Not calculated n=7697, m=1	Not calculated n=7697, m=1	Not calculated n=7698, m=0
Poultry and rabbit farming	1.01 [0.58−1.72] n=9657, m=14	0.60 [0.22−1.64] n=9667, m=4	Not calculated n=9670, m=1	Not calculated n=9670, m=1	Not calculated n=9669, m=2	1.85 [0.89−3.85] n=9663, m=8	Not calculated n=9670, m=1	Not calculated n=9671, m=0	Not calculated n=9669, m=2
Unspecified large animal farming (e.g. large dogs, zoo)	2.91 [0.93−9.08] n=1277, m=3	Not calculated n=1278, m=2	Not calculated n=1279, m=1	Not calculated n=1279, m=1	Not calculated n=1280, m=0	Not calculated n=1280, m=0	Not calculated n=1280, m=0	Not calculated n=1279, m=1	Not calculated n=1280, m=0
Stud farming	0.87 [0.36−2.15] n=6826, m=5	Not calculated n=6829, m=2	Not calculated n=6831, m=0	Not calculated n=6829, m=2	Not calculated n=6831, m=0	1.44 [0.44−4.67] n=6828, m=3	Not calculated n=6831, m=0	Not calculated n=6831, m=0	Not calculated n=6831, m=0
Pig farming	0.82 [0.33−2.03] n=3825, m=5	Not calculated n=3828, m=2	Not calculated n=3830, m=0	Not calculated n=3829, m=1	Not calculated n=3829, m=1	1.44 [0.44−4.7] n=3827, m=3	Not calculated n=3830, m=0	Not calculated n=3830, m=0	Not calculated n=3830, m=0
Ovine and caprine farming	0.75 [0.45−1.25] n=16792, m=16	0.87 [0.44−1.71] n=16799, m=9	Not calculated n=16807, m=1	0.72 [0.26−1.99] n=16804, m=4	1.3 [0.46−3.65] n=16804, m=4	0.58 [0.21−1.61] n=16804, m=4	1.79 [0.53−5.97] n=16805, m=3	Not calculated n=16807, m=1	Not calculated n=16808, m=0
Both/mixed dairy and cow farming (individuals performing both dairy and cow farming)	1.06 [0.63−1.79] n=7989, m=15	1.01 [0.47−2.17] n=7997, m=7	Not calculated n=8002, m=2	Not calculated n=8002, m=2	1.4 [0.43−4.52] n=8001, m=3	1.15 [0.46−2.85] n=7999, m=5	Not calculated n=8004, m=0	2.64 [0.79−8.79] n=8001, m=3	Not calculated n=8004, m=0
Cow farming (individuals performing only cow farming)	1.06 [0.77−1.44] n=32649, m=50	1.32 [0.87−1.99] n=32669, m=30	1.42 [0.57−3.52] n=32693, m=6	1.85 [1.11−3.11] n=32679, m=20	0.73 [0.31−1.75] n=32693, m=6	0.64 [0.33−1.26] n=32689, m=10	0.65 [0.19−2.19] n=32696, m=3	1.91 [0.79−4.56] n=32692, m=7	Not calculated n=32699, m=0
Dairy farming (individuals performing only dairy farming)	0.73 [0.55−0.97] n=48761, m=62	0.66 [0.43−1.03] n=48797, m=26	0.97 [0.45−2.08] n=48813, m=10	0.49 [0.25−0.97] n=48813, m=10	0.89 [0.43−1.83] n=48813, m=10	0.60 [0.35−1.03] n=48805, m=18	1.32 [0.56−3.11] n=48815, m=8	0.70 [0.27−1.79] n=48817, m=6	2.53 [0.85−7.48] n=48817, m=6
Unspecified specialized farming (e.g. horticulture)	Not calculated n=2231, m=2	Not calculated n=2232, m=1	Not calculated n=2233, m=0	Not calculated n=2232, m=1	Not calculated n=2233, m=0	Not calculated n=2232, m=1	Not calculated n=2233, m=0	Not calculated n=2233, m=0	Not calculated n=2233, m=0
Sylviculture/forestry	Not calculated n=339, m=0	Not calculated n=339, m=0	Not calculated n=339, m=0	Not calculated n=339, m=0	Not calculated n=339, m=0	Not calculated n=339, m=0	Not calculated n=339, m=0	Not calculated n=339, m=0	Not calculated n=339, m=0
Viticulture	1.1 [0.77−1.57] n=41921, m=49	1.36 [0.83−2.21] n=41943, m=27	1.93 [0.67−5.54] n=41964, m=6	1.56 [0.81−2.98] n=41955, m=15	0.98 [0.39−2.49] n=41963, m=7	1.11 [0.59−2.05] n=41953, m=17	0.84 [0.22−3.18] n=41967, m=3	Not calculated n=41968, m=2	Not calculated n=41970, m=0
Wheat and industrial grower (crop farming)	1.83 [1.43−2.36] n=102127, m=113	1.76 [1.22−2.53] n=102185, m=55	1.85 [0.82−4.15] n=102229, m=11	1.98 [1.21−3.23] n=102208, m=32	1.33 [0.70−2.52] n=102223, m=17	2.02 [1.29−3.17] n=102204, m=36	2.53 [1.13−5.69] n=102229, m=11	1.67 [0.73−3.79] n=102230, m=10	0.93 [0.26−3.25] n=102236, m=4
Garden center	Not calculated n=1356, m=2	Not calculated n=1356, m=2	Not calculated n=1358, m=0	Not calculated n=1356, m=2	Not calculated n=1358, m=0	Not calculated n=1358, m=0	Not calculated n=1358, m=0	Not calculated n=1358, m=0	Not calculated n=1358, m=0
Fruit arboriculture	1.3 [0.68−2.47] n=7639, m=10	Not calculated n=7647, m=2	Not calculated n=7649, m=0	Not calculated n=7648, m=1	Not calculated n=7648, m=1	2.33 [0.99−5.44] n=7643, m=6	Not calculated n=7648, m=1	Not calculated n=7648, m=1	Not calculated n=7649, m=0
Truck farming, floriculture/flower−growing	1.41 [0.85−2.34] n=12655, m=17	1.5 [0.72−3.11] n=12664, m=8	Not calculated n=12670, m=2	1.39 [0.49−3.89] n=12668, m=4	Not calculated n=12670, m=2	1.15 [0.45−2.9] n=12667, m=5	Not calculated n=12671, m=1	Not calculated n=12670, m=2	Not calculated n=12671, m=1

Fig. S7: Results from sensitivity analysis (age as continuous variable) - Agricultural practices and risks of CNS tumors for women, TRACTOR, France, 2002-2016

Note: n: number of exposed farm managers, m: number of exposed cases, NC: not calculated, CNS: central nervous system, C70: malignant neoplasm of meninges, D32: benign neoplasm of meninges, D42: neoplasm of uncertain or unknown behavior of meninges, 95% CI: 95% confidence interval.

Hazard ratios were estimated by Cox models with time to first CNS tumor insurance declaration as the underlying timescale, when the number of exposed cases was sufficient ($m \geq 3$), age, first year of the farm's establishment, farm surface, earnings, number of associates, unemployment status, total number of farms, family status, partner work status, farm location, number of comorbidities and having a secondary activity.

Cells with red background refer to increased risks of CNS tumors, while cells with green background refer to decreased risk of CNS tumors.

	All CNS tumors	All meningiomas	C70	D32	D42	C71	D33	D43	C72
Rural craftsperson	Not calculated n=6782, m=0	Not calculated n=6782, m=0	Not calculated n=6782, m=0	Not calculated n=6782, m=0	Not calculated n=6782, m=0	Not calculated n=6782, m=0	Not calculated n=6782, m=0	Not calculated n=6782, m=0	Not calculated n=6782, m=0
Company representative/authorized representative	Not calculated n=408, m=1	Not calculated n=409, m=0	Not calculated n=409, m=0	Not calculated n=409, m=0	Not calculated n=409, m=0	Not calculated n=408, m=1	Not calculated n=409, m=0	Not calculated n=409, m=0	Not calculated n=409, m=0
Gardening, landscaping and reforestation companies	0.75 [0.48−1.17] n=42558, m=21	Not calculated n=42577, m=2	Not calculated n=42578, m=1	Not calculated n=42578, m=1	Not calculated n=42579, m=0	0.75 [0.42−1.36] n=42567, m=12	1.53 [0.46−5.01] n=42576, m=3	1.21 [0.32−4.53] n=42575, m=4	Not calculated n=42579, m=0
Agricultural work companies	1.33 [0.73−2.43] n=12556, m=11	2.59 [0.94−7.07] n=12563, m=4	Not calculated n=12567, m=0	Not calculated n=12566, m=1	5.05 [1.54−16.6] n=12564, m=3	0.84 [0.31−2.26] n=12563, m=4	Not calculated n=12565, m=2	Not calculated n=12566, m=1	Not calculated n=12567, m=0
Fixed sawmill	Not calculated n=687, m=0	Not calculated n=687, m=0	Not calculated n=687, m=0	Not calculated n=687, m=0	Not calculated n=687, m=0	Not calculated n=687, m=0	Not calculated n=687, m=0	Not calculated n=687, m=0	Not calculated n=687, m=0
Wood production	1.17 [0.62−2.2] n=10177, m=10	Not calculated n=10185, m=2	Not calculated n=10187, m=0	Not calculated n=10186, m=1	Not calculated n=10186, m=1	1.31 [0.58−2.95] n=10181, m=6	Not calculated n=10187, m=0	Not calculated n=10186, m=1	Not calculated n=10186, m=1
Salt marsh	Not calculated n=673, m=0	Not calculated n=673, m=0	Not calculated n=673, m=0	Not calculated n=673, m=0	Not calculated n=673, m=0	Not calculated n=673, m=0	Not calculated n=673, m=0	Not calculated n=673, m=0	Not calculated n=673, m=0
Unspecified and mixed farming (e.g. polyculture, mixed farming)	1.1 [0.84−1.45] n=83710, m=81	0.67 [0.33−1.35] n=83780, m=11	0.67 [0.16−2.77] n=83788, m=3	0.33 [0.09−1.19] n=83788, m=3	1.23 [0.46−3.29] n=83785, m=6	1.29 [0.91−1.83] n=83738, m=53	0.99 [0.39−2.5] n=83783, m=8	1.64 [0.76−3.52] n=83781, m=10	Not calculated n=83790, m=1
Shellfish farming	Not calculated n=2682, m=2	Not calculated n=2684, m=0	Not calculated n=2684, m=0	Not calculated n=2684, m=0	Not calculated n=2684, m=0	Not calculated n=2682, m=2	Not calculated n=2684, m=0	Not calculated n=2684, m=0	Not calculated n=2684, m=0
Training, dressage, riding clubs	1.1 [0.49−2.47] n=7218, m=6	Not calculated n=7223, m=1	Not calculated n=7224, m=0	Not calculated n=7224, m=0	Not calculated n=7223, m=1	0.96 [0.30−3.01] n=7221, m=3	Not calculated n=7224, m=0	Not calculated n=7222, m=2	Not calculated n=7224, m=0
Unspecified small animal farming (e.g. frogs, snails, bees)	1.11 [0.45−2.67] n=10355, m=5	Not calculated n=10359, m=1	Not calculated n=10360, m=0	Not calculated n=10360, m=0	Not calculated n=10359, m=1	1.15 [0.36−3.59] n=10357, m=3	Not calculated n=10360, m=0	Not calculated n=10359, m=1	Not calculated n=10360, m=0
Poultry and rabbit farming	0.79 [0.43−1.45] n=14894, m=11	Not calculated n=14903, m=2	Not calculated n=14905, m=0	Not calculated n=14904, m=1	Not calculated n=14904, m=1	0.58 [0.24−1.42] n=14900, m=5	Not calculated n=14903, m=2	Not calculated n=14903, m=2	Not calculated n=14905, m=0
Unspecified large animal farming (e.g. large dogs, zoo)	Not calculated n=1382, m=1	Not calculated n=1383, m=0	Not calculated n=1383, m=0	Not calculated n=1383, m=0	Not calculated n=1383, m=0	Not calculated n=1382, m=1	Not calculated n=1383, m=0	Not calculated n=1383, m=0	Not calculated n=1383, m=0
Stud farming	Not calculated n=8809, m=1	Not calculated n=8809, m=1	Not calculated n=8810, m=0	Not calculated n=8809, m=1	Not calculated n=8810, m=0	Not calculated n=8810, m=0	Not calculated n=8810, m=0	Not calculated n=8810, m=0	Not calculated n=8810, m=0
Pig farming	1.95 [1.21−3.14] n=9540, m=19	2.27 [0.79−6.44] n=9555, m=4	Not calculated n=9558, m=1	Not calculated n=9559, m=0	6.03 [1.75−20.8] n=9556, m=3	2.27 [1.29−3.99] n=9545, m=14	Not calculated n=9559, m=0	Not calculated n=9559, m=0	Not calculated n=9558, m=1
Ovine and caprine farming	1.2 [0.82−1.76] n=30249, m=29	1.58 [0.72−3.44] n=30271, m=7	Not calculated n=30277, m=1	Not calculated n=30276, m=2	2.26 [0.80−6.36] n=30274, m=4	1.33 [0.83−2.13] n=30259, m=19	Not calculated n=30277, m=1	0.91 [0.28−2.97] n=30275, m=3	Not calculated n=30278, m=0
Both/mixed dairy and cow farming (individuals performing both dairy and cow farming)	0.81 [0.52−1.26] n=22704, m=21	2.22 [1.2−4.09] n=22713, m=12	1.65 [0.49−5.56] n=22722, m=3	1.16 [0.35−3.79] n=22722, m=3	4.34 [1.76−10.7] n=22719, m=6	0.39 [0.17−0.87] n=22719, m=6	Not calculated n=22724, m=1	Not calculated n=22723, m=2	Not calculated n=22725, m=0
Cow farming (individuals performing only cow farming)	0.86 [0.66−1.12] n=77444, m=71	0.94 [0.54−1.65] n=77499, m=16	0.87 [0.29−2.61] n=77511, m=4	1.42 [0.66−3.05] n=77506, m=9	0.49 [0.14−1.67] n=77512, m=3	0.68 [0.47−0.98] n=77480, m=35	0.59 [0.20−1.7] n=77511, m=4	2.11 [1.11−4.02] n=77501, m=14	Not calculated n=77513, m=2
Dairy farming (individuals performing only dairy farming)	1.12 [0.90−1.38] n=109759, m=124	0.67 [0.40−1.11] n=109864, m=19	1.15 [0.48−2.77] n=109875, m=8	0.80 [0.38−1.66] n=109873, m=10	Not calculated n=109881, m=2	1.32 [1.02−1.71] n=109802, m=81	1.24 [0.55−2.75] n=109875, m=8	0.77 [0.38−1.58] n=109873, m=10	1.77 [0.67−4.6] n=109876, m=7
Unspecified specialized farming (e.g. horticulture)	Not calculated n=3933, m=2	Not calculated n=3934, m=1	Not calculated n=3935, m=0	Not calculated n=3934, m=1	Not calculated n=3935, m=0	Not calculated n=3935, m=0	Not calculated n=3934, m=1	Not calculated n=3935, m=0	Not calculated n=3935, m=0
Sylviculture/forestry	Not calculated n=1645, m=2	Not calculated n=1647, m=0	Not calculated n=1647, m=0	Not calculated n=1647, m=0	Not calculated n=1647, m=0	Not calculated n=1645, m=2	Not calculated n=1647, m=0	Not calculated n=1647, m=0	Not calculated n=1647, m=0
Viticulture	1.12 [0.82−1.51] n=76543, m=64	1.49 [0.75−2.94] n=76594, m=13	Not calculated n=76607, m=0	2.16 [0.82−5.66] n=76600, m=7	1.71 [0.61−4.81] n=76601, m=6	1.08 [0.72−1.62] n=76571, m=36	1.5 [0.63−3.54] n=76598, m=9	0.91 [0.35−2.37] n=76601, m=6	Not calculated n=76606, m=1
Wheat and industrial grower (crop farming)	1.4 [1.13−1.74] n=203453, m=145	1.59 [0.99−2.54] n=203566, m=32	1.28 [0.50−3.27] n=203590, m=8	1.88 [0.91−3.87] n=203584, m=14	1.72 [0.78−3.76] n=203586, m=12	1.33 [1−1.78] n=203518, m=80	2.29 [1.11−4.7] n=203580, m=18	0.79 [0.38−1.65] n=203587, m=11	2.18 [0.78−6.03] n=203591, m=7
Garden center	Not calculated n=3752, m=1	Not calculated n=3753, m=0	Not calculated n=3753, m=0	Not calculated n=3753, m=0	Not calculated n=3753, m=0	Not calculated n=3753, m=0	Not calculated n=3752, m=1	Not calculated n=3753, m=0	Not calculated n=3753, m=0
Fruit arboriculture	1.49 [0.91−2.42] n=16420, m=17	Not calculated n=16436, m=1	Not calculated n=16437, m=0	Not calculated n=16436, m=1	Not calculated n=16437, m=0	1.25 [0.61−2.55] n=16429, m=8	Not calculated n=16435, m=2	4.08 [1.57−10.6] n=16432, m=5	Not calculated n=16435, m=2
Truck farming, floriculture/flower−growing	1.46 [0.98−2.19] n=28826, m=27	1.96 [0.83−4.6] n=28847, m=6	Not calculated n=28852, m=1	3.08 [1.04−9.11] n=28849, m=4	Not calculated n=28852, m=1	1.03 [0.57−1.88] n=28841, m=12	Not calculated n=28851, m=2	1.81 [0.62−5.19] n=28849, m=4	6.75 [1.81−25.2] n=28850, m=3

Fig. S8: Results from sensitivity analysis (age as continuous variable) - Agricultural practices and risks of CNS tumors for men, TRACTOR, France, 2002-2016

Note: n: number of exposed farm managers, m: number of exposed cases, NC: not calculated, CNS: central nervous system, C70: malignant neoplasm of meninges, D32: benign neoplasm of meninges, D42: neoplasm of uncertain or unknown behavior of meninges, 95% CI: 95% confidence interval.

Hazard ratios were estimated by Cox models with time to first CNS tumor insurance declaration as the underlying timescale, when the number of exposed cases was sufficient ($m \geq 3$), age, first year of the farm's establishment, farm surface, earnings, number of associates, unemployment status, total number of farms, family status, partner work status, farm location, number of comorbidities and having a secondary activity.

Cells with red background refer to increased risks of CNS tumors, while cells with green background refer to decreased risk of CNS tumors.
